# Freeze-Driven Synthesis of DNA Hairpin-Conjugated Gold Nanoparticle Biosensors for Dual-Mode Detection

**DOI:** 10.1021/acsabm.4c00069

**Published:** 2024-04-17

**Authors:** Angela Michelle San Juan, Siddhant Jaitpal, Ka Wai Ng, Cecilia Martinez, Sayantan Tripathy, Christian Phillips, Gerard L Coté, Samuel Mabbott

**Affiliations:** † Department of Biomedical Engineering, Texas A&M University, College Station, Texas 77843-3120, United States; ‡ Center for Remote Health Technologies & Systems, Texas A&M Engineering Experiment Station, College Station, Texas 77845-3424, United States

**Keywords:** freeze-assisted, DNA Hairpins, gold nanoparticles, surface-enhanced Raman scattering, fluorescence, biosensing, surface functionalization

## Abstract

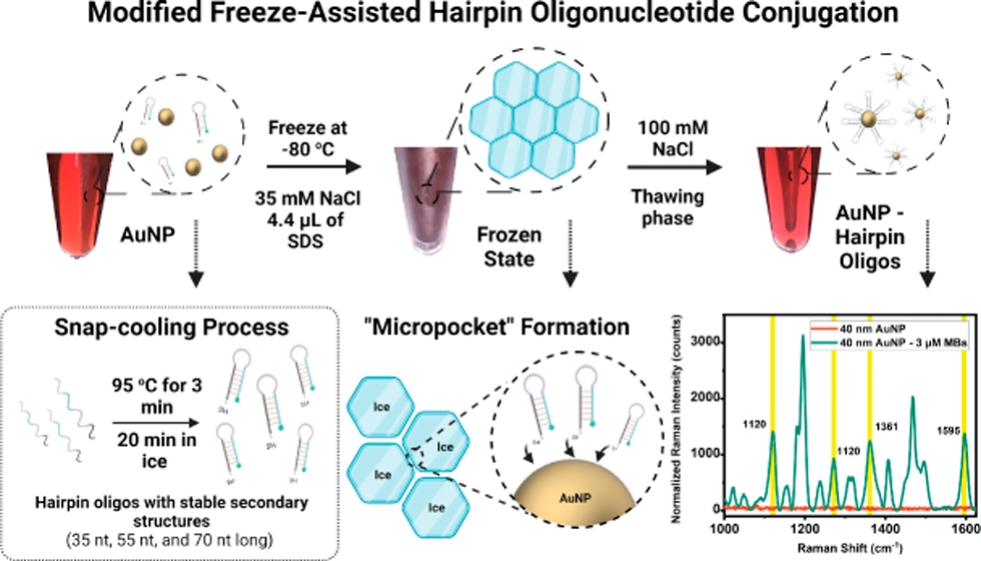

Freeze-based immobilization of deoxyribonucleic acid (DNA) oligonucleotides on gold nanoparticles (AuNPs) is highly efficient for single-stranded oligonucleotides but typically does not accommodate structures such as snap-cooled DNA hairpins (Sc-HPs) and snap-cooled molecular beacons (Sc-MBs) frequently used for biorecognition applications. Recognizing this limitation, we have developed a modified, freeze-based technique specifically designed to enable the adsorption of such hairpin oligonucleotides onto AuNP surfaces while ensuring that they retain their biosensing capabilities. Successful hairpin oligonucleotide conjugation of varying lengths to a wide range of AuNP diameters was corroborated by dynamic light scattering, ζ-potential, and UV–vis spectrophotometry. Moreover, we conducted a thorough evaluation of this modified method, confirming the retention of the sensing functions of Sc-HPs and Sc-MBs. This advancement not only offers a more efficient route for DNA hairpin conjugation but also elucidates the underlying biorecognition functions, with implications for broader applications in molecular diagnostics.

## Introduction

The field of nanomedicine has seen tremendous advancements in recent years, thanks in large part to the emergence of novel biofunctionalization techniques for the strategic attachment of biologically active molecules to surfaces.^1−3^ Biofunctionalization is critical for increasing the efficacy and targeting ability of gold nanoparticles (AuNPs) in a diverse array of biomedical applications including targeted drug delivery,^4^ diagnostic imaging,^5^ biosensing,^6^ and photothermal therapy.^7^ Of particular note is the functionalization of AuNPs with nucleic acids, which facilitates highly precise identification of pathogens, detection of genetic mutations, and diagnosis of diseases.^8,9^ Furthermore, nucleic acid-functionalized AuNPs offer unprecedented opportunities for gene silencing,^10^ cellular tracking,^11^ and the creation of self-assembled nanostructures.^12^ Despite the relative simplicity of the nucleic acid building blocks from which the attached sequences are constructed, their diverse arrangements can be used to generate complex structures capable of being applied to specific biomedical tasks. Consider, for instance, aptamers, which are single-stranded nucleic acid molecules commonly constructed from either DNA or RNA, that can bind to proteins or small molecules with high affinity.^13^ Their binding ability arises from their unique three-dimensional configurations, including stem-loop structures, hairpins, bulges, internal loops, junctions, and pseudoknots.^14,15^ Aptamers spontaneously fold into these secondary structures under appropriate conditions, a process driven by the thermodynamics of nucleotide interactions.^14,15^ The resulting structure is a delicate balance of hydrogen bonding among nucleotides, base stacking interactions, and the overall thermodynamic stability of the conformation.^16^ This secondary structure is crucial for the aptamer’s capacity to recognize and bind its target molecule.^16^ The attachment of DNA hairpin sequences to AuNPs has garnered significant attention within the biosensing community for the targeting of nucleic acid biomarkers.^17−19^ Utilizing DNA hairpins for biomarker recognition presents numerous benefits over linear sequences, such as enhanced specificity, greater stability, minimized nonspecific binding, dynamic sensing, and flexible design possibilities.^17−19^

Considering these benefits, it is essential to ensure that any biofunctionalization strategy used to attach these hairpin sequences to AuNPs preserves the structural integrity of the hairpins as their secondary structure is pivotal to their sensing performance. Researchers predominantly rely on the robust covalent bonds formed between AuNPs and thiolated molecules, taking advantage of the affinity between thiols and gold. However, alternative strategies for functionalizing AuNPs also exist. These include the utilization of bifunctional linkers, such as derivatives of polyethylene glycol (PEG), the employment of electrostatic interactions involving cationic agents like poly l-lysine or chitosan, the application of high-affinity streptavidin–biotin binding, the incorporation of click chemistry approaches like copper-catalyzed azide–alkyne cycloaddition, the exploitation of photochemically facilitated attachments, and the implementation of enzymatic ligation techniques.^17−19^

Recent advances in the field of DNA-AuNP conjugation have broadened the spectrum of the available strategies. An innovative method is the freeze-assisted DNA-AuNP conjugation, allowing rapid DNA adsorption onto AuNPs in just 15 min.^20−22^ This technique builds upon the previously documented freeze-driven ssDNA adsorption by Liu and Liu where thiolated probes were utilized to immobilize on the AuNP surface by exploiting micropockets that emerge upon the formation of ice crystals.^20−22^ Unlike many conventional thiol-based techniques, Lui’s freeze-assisted method successfully avoids the use of tris(2-carboxyethyl)phosphine (TCEP) and dithiothreitol (DTT) as reducing agents for breaking disulfide bonds. Subsequent research found that freeze-assisted conjugation causes the oligonucleotides to align laterally and stretch, exposing the thiol group more prominently. This improved the density, stability, and activity of the DNA attached to the AuNPs. Currently, the entire body of reported work using freeze-assisted methodologies is focused on attaching linear nucleic acid sequences to the surfaces of AuNPs. Until now, to the best of our knowledge, studies have not explored freeze-assisted techniques for conjugating AuNPs with hairpin probes. The primary concern is whether this technique maintains the hairpins’ sensory capabilities. In this work, we evaluate the effectiveness of the freeze-assisted method for hairpin attachment. Using a DNA analogue of micro-RNA-574–5p (miR-574–5p) as a benchmark, we affirm that hairpins retain their sensing potential postconjugation with AuNPs through this method. Further, when these hairpins are integrated with a fluorophore/Raman reporter dye, they showcase dual fluorescence and SERS detection, highlighting their multifaceted utility in identifying biomarkers, notably, miR-574–5p, linked to thoracic aortic aneurysm.

## Results and Discussion

### Freeze-Driven Snap-Cooled DNA Hairpin (Sc-HP) and Snap-Cooled Molecular Beacon (Sc-MB) Conjugation

The freeze-assisted DNA oligonucleotide labeling strategy investigated by Liu et al. is a versatile strategy that only requires a refrigerator or an ultralow-temperature freezer.^20,21^ This technique allows for efficient immobilization of oligonucleotides for approximately 1 h, and the time can be further reduced to 15 min when employing an ultralow-temperature freezer. This method was achievable due to the formation of micropockets when ice crystals are formed, forcing nonwater species such as DNA oligonucleotides, AuNPs, and salts into these gaps.^20,21^ This eutectic phase is composed of a saturated salt concentration region, forcing the interaction between the DNA oligonucleotides and AuNPs and overcoming the electrostatic repulsions.^20,21^ Additionally, the authors also reported stretching and aligning upon freezing, which could elucidate the fast DNA adsorption.^23^ It was noted that the most suitable sequences should contain no stable secondary structures for efficient labeling.^20,21^

**Figure 1:**
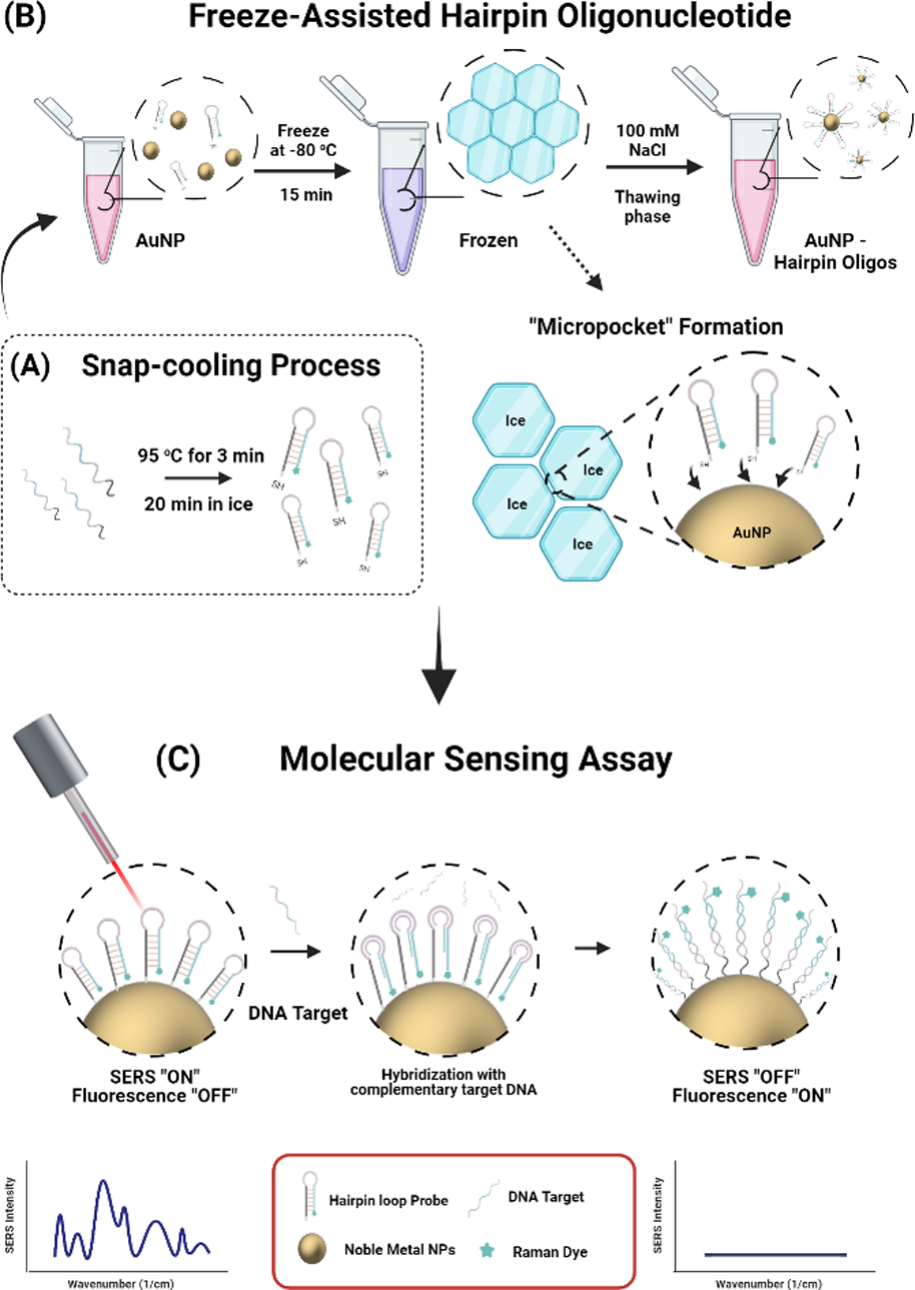
(A) Demonstrates the snap-cooling process by heating the Sc-HPs and Sc-MBs at 95 °C for 3 min and immediately cooling the samples in ice for 20 min. (B) Schematic representation illustrating the freeze-assisted hairpin oligonucleotide deposition on the bare AuNP surface. (C) Depicts a molecular sensing assay involving the addition of a DNA target sequence which causes the fluorophore/Raman reporter molecule to displace away from the surface. This results in a SERS “ON” to “OFF” configuration while the reverse is seen for fluorescence as the AuNPs serves as quenchers.

Current methodologies for immobilizing DNA oligonucleotides typically do not accommodate structures with inherent secondary structures such as Sc-HPs and Sc-MBs. Recognizing this limitation, we have developed a modified freeze-assisted technique specifically designed to enable the adsorption of such oligonucleotides onto surfaces. Moreover, we have conducted a thorough evaluation of this technique, confirming the retention of the sensing functions of Sc-HPs and Sc-MBs. Figure 1 demonstrates the modified freeze-assisted conditions to immobilize the Sc-HPs and Sc-MBs. Surface-enhanced Raman spectroscopy (SERS) and fluorescence measurements were utilized to validate the sensing functionalities of the immobilized Sc-HPs and Sc-MBs, as illustrated in Figure 1C.

### Native PAGE Under Freeze-Assisted Immobilization Conditions

Using NUPACK software for nucleic acid analysis and simulations, we designed a hairpin sequence (sensing strand) tailored for specific binding to the miR-574–5p model target. Our top candidate, a 35-base sequence, showcases a 23-base single-stranded DNA (ssDNA) loop linked to a double stranded DNA (dsDNA) stem region. With a change in Gibbs free energy (Δ*G*) of −4.71 kcal/mol, this sequence demonstrates a thermodynamically stable secondary structure that forms spontaneously at room temperature (25 °C, Figure S2). When simulated at physiological temperature (37 °C), the binding interaction between the hairpin and the target sequence yielded an Δ*G* of −32.62 kcal/mol, highlighting a robust and energetically favorable association between them (Figure S3). To allow for gold nanoparticle anchoring, a thiol modification to the designed hairpins (Sc-HPs) was added to the sequence on its 5′ end.

**Figure 2:**
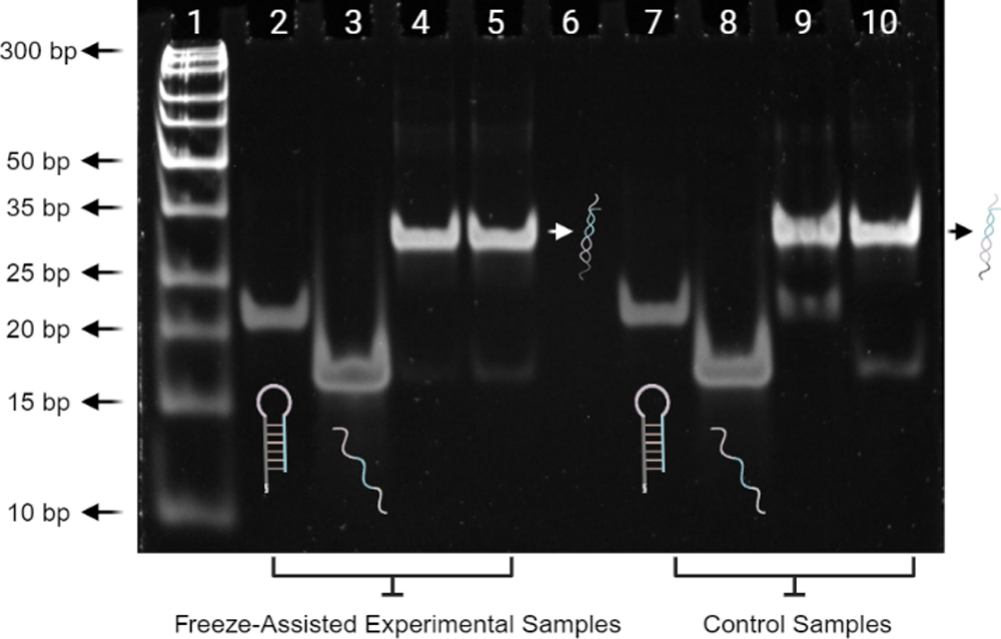
15% Native polyacrylamide gel electrophoresis results for Sc-HP hybridization with a DNA target sequence at a 1:1 molar ratio. Lane 1:20 base pair (bp) DNA ladder; Lane 2: Sc-HPs that have undergone the freeze-assisted conditions; Lane 3: DNA target sequence; Lane 4–5:1:1 molar ratio of Sc-HPs that have undergone the freeze-assisted condition and DNA target sequence; Lane 7: Sc-HPs control samples; Lane 8: DNA target sequence; Lane 9–10:1:1 molar ratio of Sc-HPs and DNA target sequence.

Native gel electrophoresis was employed to verify that the hairpins retained their target-binding ability post-freeze-assisted functionalization. Figure 2 illustrates the electrophoretic profile: lane 1 displays the ultralow range DNA ladder (10–300 base pair (bp)), while lanes 2 and 7 feature the treated Sc-HPs and their control counterparts, respectively. Lanes 3 and 8 contain the target DNA sequence. In lanes 2 and 3, as well as 7 and 8, two separate bands appear, corresponding to the Sc-HPs and the target DNA sequence, with their distinct molecular weights (Sc-HPs at 10,782 g/mol and the DNA target at 7227.7 g/mol). This discrepancy in weight accounts for the more pronounced mobility shift in the target DNA sequence. Lanes 4 and 5 show a combined sample of treated Sc-HPs and target DNA at a 1:1 molar ratio, incubated at 37 °C for 3 h, where hybridization was inferred from the minimal presence of free hairpins or target sequences. In contrast, lanes 9 and 10, which held samples not subjected to the freeze-assisted process, exhibited less distinct bands at the lower molecular weight region, near the 20 and 15 bp markers, indicating the presence of unhybridized hairpins and target sequences. These findings affirm that Sc-HPs maintain their structural integrity and functional sensitivity even after freeze-assisted processing.

### Confirming the Functionalization of Sc-HPs on AuNPs

Upon confirming the spontaneous formation of the hairpin’s secondary structure and its hybridization ability with the target sequence through electrophoresis, we then proceeded to functionalize the sequences. Specifically, we designed a sensing strand akin to a molecular beacon (Sc-MB), which was modified by attaching a dithiol group to its 5′-end and conjugating a Cyanine5 (Cy5) fluorescent label at the 3′-end. Our rationale was that the hairpin sequences, through dithiol modification, could anchor onto the AuNP surface. Given that AuNPs can function as SERS signal transducers, we posited that variations in SERS intensity, reflecting the distance of the Cy5 tag from the nanoparticle, could verify the hairpin’s unfolding in the target’s presence. This would further assess if the hairpin’s sensing capabilities remained intact under freeze-assisted bioconjugation protocols. Additionally, the functionality of the hairpin would be confirmed by an increase in fluorescence from the AuNP equipped with the molecular beacon in the presence of the target, since it is expected that the Cy5 molecule will be far enough from the AuNP surface not to have its fluorescence signal quenched.

Prior research carried out on the freeze-assisted bioconjugation of linear DNA sequences to an AuNP surface via dithiol anchoring indicated that reduction of the dithiols using TCEP was not necessary; however, when we attempted to attach, snap-cooled, non-TCEP treated Sc-HPs (0.5–9 μM) to our AuNPs (20 and 40 nm diameter, optical density per milliliter (OD) 10, we observed that the AuNPs became irreversibly aggregated after thawing, indicating that the attachment of the Sc-HPs was unsuccessful. The UV–vis spectra in Figure S4 show the formation of absorbance peaks >600 nm in both sets of thawed AuNPs (20 and 40 nm) incubated with dithiol Sc-HPs, which indicates the formation of nanoparticle aggregates. To overcome this instability issue, the dithiol sequence was pretreated with tris(2-carboxyethyl) phosphine hydrochloride (TCEP) to reduce the dithiols before snap-cooling; furthermore, sodium dodecyl sulfate (SDS), which has been reported to decrease the tendency for AuNPs to aggregate and coalesce, was added to the AuNPs. To test the assumption that SDS addition would help increase the stability of the AuNPs, we added sodium chloride (NaCl) to suspensions containing 40 nm AuNPs (with and without SDS). The UV–vis plots in Figure S5 show that in the absence of SDS, the AuNPs destabilize, indicated by a flat and broadened spectrum when the NaCl concentration increases above 30 mM (Figure S4A); however, with the addition of SDS to the AuNP suspension that can be seen to preserve the stability when up to 60 mM of NaCl is added (Figure S5B), the normalized spectra in Figure S4C provide further indication that no change in the extinction spectrum is observed when compared to the AuNP control spectrum (λ_max_ = 520 nm).

**Figure 3:**
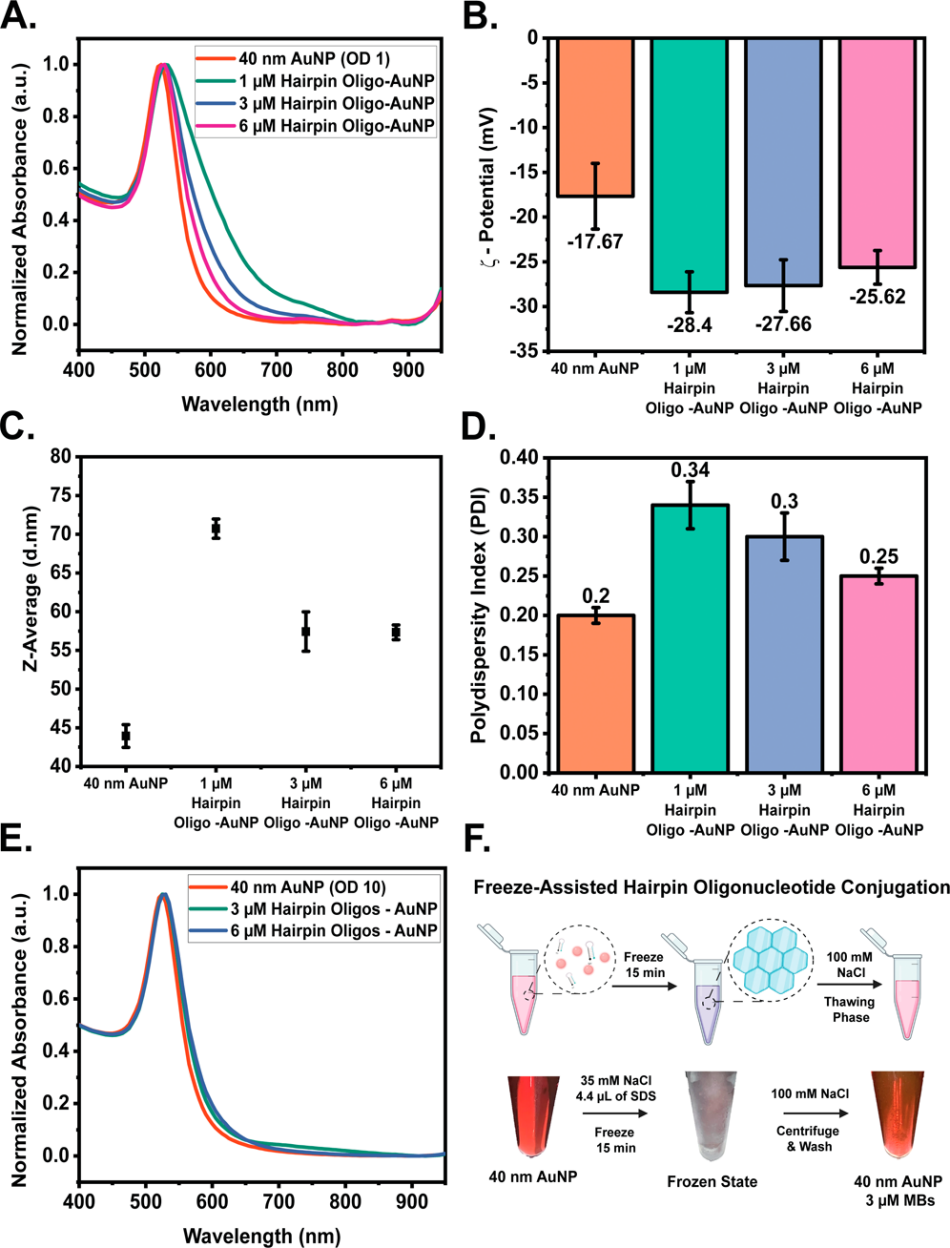
(A) Effect of initial snap-cooled hairpin DNA concentration with a fixed AuNP concentration. (B–D) Hydrodynamic diameter, ζ-potential measurements, and polydispersity index confirming successful DNA hairpin immobilization on the AuNPs. (E). Schematic and images illustrating the freeze-assisted immobilization as well as the color change of AuNPs after a freeze/thawing cycle. (F). UV–vis measurements verify optical stability of 40 nm AuNP OD 10 in the presence of various concentrations of DNA hairpins after the freeze-assisted immobilization.

In our subsequent experiment, the freeze-assisted hairpin bioconjugation approach was applied using 40 nm AuNPs suspended in solutions containing NaCl, SDS, and varying concentrations of Sc-HP (1, 3, and 6 μM). Successful attachment of the Sc-HPs to the AuNPs was confirmed by the UV–vis spectra shown in Figure 3A, where an average shift of 5 nm to higher wavelength was observed in the λ_max_ for the Sc-HP conjugated AuNPs compared to the bare AuNP control which is caused by the change in refractive index. The samples modified with 1 and 3 μM Sc-HPs exhibited significant peak broadening, suggesting changes in nanoparticle morphology due to their instability. At an initial 6 μM Sc-HPs, the spectra and polydispersity index (PDI) from 0.2 to 0.25 illustrated a more promising Sc-HPs - AuNP conjugate formation. This formation of Sc-HPs – AuNP conjugates (1, 3, and 6 μM) were all validated using ζ - potential and hydrodynamic diameter analysis. ζ - potential analyses (Figure 3B) corroborated these findings, with the 40 nm AuNP being moderately stable (−17.67 mV). However, an increase in negative ζ potentials was observed in the AuNP-Sc-HP batches, underscoring the augmented surface charge attributed to oligonucleotide binding. Using dynamic light scattering (DLS, Figure 3C), we also observed an increase in hydrodynamic diameter upon Sc-HP functionalization. The uniformity of the particles was assessed by measuring the PDI (Figure 3D). While the unmodified 40 nm AuNP could be considered monodispersed (PDI of 0.2), an increase in PDI values was observed among the 1 and 3 μM modified Sc-HP functionalized AuNPs. However, the AuNPs incubated with 6 μM Sc-HP exhibited only a small increase in comparison to the other experimental samples, which suggests that a higher concentration of Sc-HPs > 6 μM allows for the stable and monodisperse functionalization of unmodified AuNPs.

Interestingly, when we increased the AuNP concentration to OD 10 and incubated them with the Sc-HPs (3 and 6 μM, Figure 3E), we still observed a slight shift in the λ_max._ However, the peak broadening was less prominent, suggesting that the bioconjugation had been successful and that the attachment process did not induce aggregation of the particles. A visual example of the AuNPs before and after functionalization with the Sc-HPs is displayed in Figure 3F. Attachment of oligonucleotides is known to enhance the stability of AuNPs under high salt concentrations. Therefore, to further evaluate the immobilization of the Sc-HP sequences, varying NaCl concentrations from 0 to 1000 mM NaCl were spiked into the hairpin functionalized AuNPs. To observe the protective properties of the hairpin probe sequences, UV–vis measurements were conducted. Interestingly, as shown in Figure S6C, Sc-HPs in nuclease-free water observed slight broadening. However, once 100 mM NaCl was added to the solution, the Sc-HP AuNPs stabilized. The conjugates remained stable in the presence of 100 to 750 mM NaCl. This finding confirms the protective properties of the immobilized Sc-HPs as the presence of >60 mM NaCl typically aggregates bare AuNPs (Figure S5A). Furthermore, only spectral broadening was observed at NaCl concentrations >750 mM NaCl, as seen in Figure S6C.

The loading of the Sc-HPs was evaluated using a nanodrop instrument. It was found that 25% of the initial 3 μM Sc-HPs and 18.6% of the initial 6 μM Sc-HPs added in the reaction mixture were successfully loaded on the nanoparticles after the freeze-assisted immobilization (Figure S7). We further evaluated this technique to be applied to snap-cooled hairpins with longer sequences (>35 nucleotides (nt)) and other AuNP diameters, a 55 nt and 70 nt long sequences were further designed and briefly evaluated. The UV–visible spectra did not display any significant red-shifting across the experimental samples (Figures S9 and S10). Complementary dynamic light scattering data revealed an increase in hydrodynamic size (Table S10), while ζ-potential measurements indicated a decrease (Figure S11), both consistent with successful oligonucleotide attachment.

### Confirming the Functionalization of Sc-MBs on AuNPs

**Figure 4:**
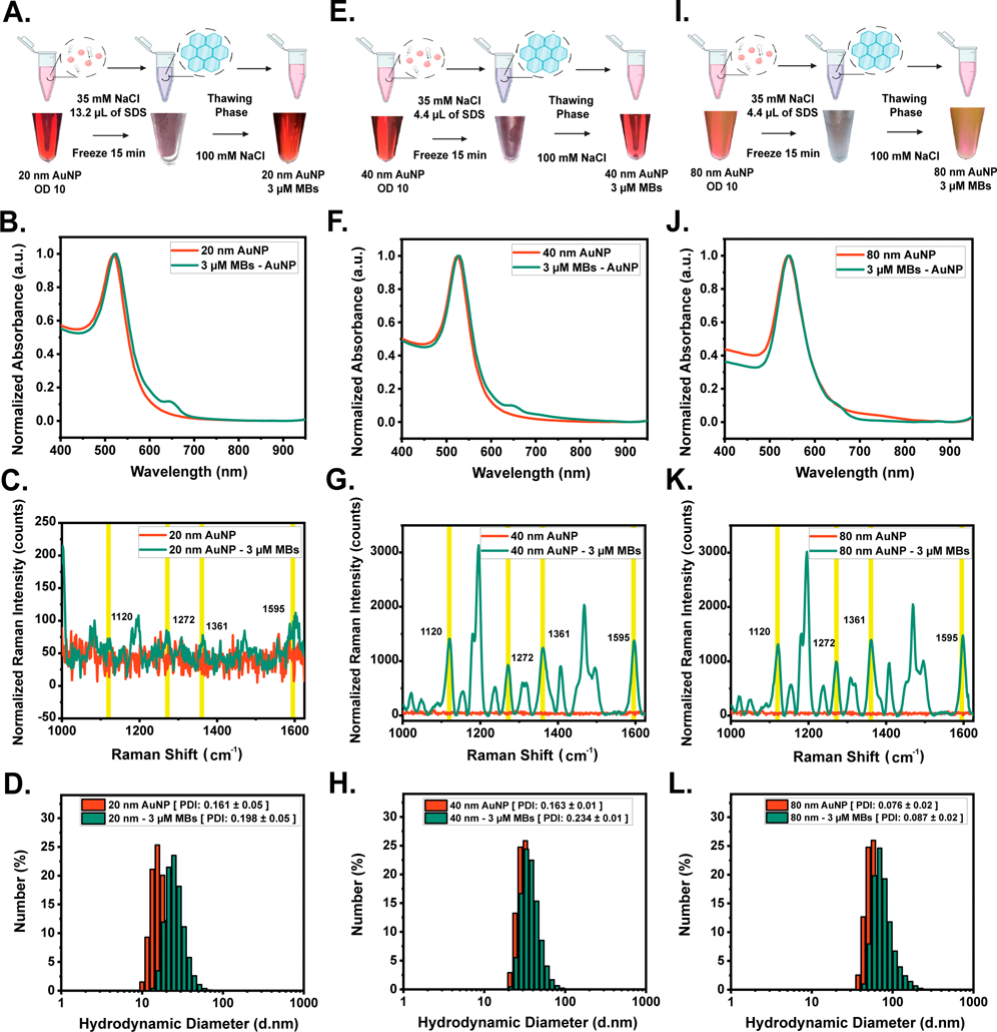
Effect of AuNP size with fixed initial snap-cooled hairpin molecular beacons concentration. (A, E, and I) Color change of 20, 40, and 80 nm AuNPs (OD 10) in the presence of 3 μM Sc-MBs after the freeze-assisted immobilization method. (B, F, J) UV–vis spectra of the 20, 40, and 80 nm AuNP, and 3 μM Sc-MBs immobilized 20 nm AuNPs after washing. (C, G, K) SERS measurements of 20, 40, and 80 nm AuNP, and 3 μM Sc-MBs immobilized 20 nm AuNPs after washing. (D, H, L) Hydrodynamic size of 20, 40, and 80 nm AuNP, and 3 μM Sc-MBs immobilized 20 nm AuNPs.

Given that our findings demonstrated that the incubation of 3 μM hairpins with the AuNPs (OD 10) yielded stable bioconjugate nanoparticles showcasing optimal hairpin loading, we proceeded to employ these in subsequent experiments. To further evaluate whether the freeze-assisted method preserved the SERS sensing capabilities of the hairpin sequences, Sc-MBs equipped with a 5′ dithiol functional group and a 3′ Cy5 dye, functioning as both a Raman reporter molecule and a fluorophore, were employed. Furthermore, to evaluate the SERS effect based on AuNP size and to further assess the viability of this approach, we utilized AuNPs of varying diameters, specifically 20, 40, and 80 nm. Figure 4 systematically details the procedure and outcomes of conjugating Sc-MBs with AuNPs of different sizes using the freeze-assisted method. Experimental observations for each set of AuNPs, 20, 40, and 80 nm, are shown in Figure 4A,E,I, respectively.

The images show the color of the AuNPs before and after they undergo freeze-assisted biofunctionalization as well as an image of each sample during its frozen state. No visible difference can be observed between the beginning and final states of the particles; however, it is worth noting that a high concentration of SDS was added to the 20 nm AuNPs to help improve their stability prior to freezing them. The UV–vis measurements (Figure 4B,F,J) corroborate the successful binding of the Sc-MBs on the surface of the bare AuNPs as all particles exhibited a slight redshift at the formation of a new absorbance peak at ∼650 nm corresponding to the Cy5 modification. The UV–vis measurements of the Sc-MB immobilized AuNPs, following the freeze-assisted method, both pre- and post-washing with the chosen hybridization buffer, are depicted in Figure S8. Specifically, Figure S12A shows the UV–vis spectra with a pronounced absorbance peak at ∼650 nm. This peak is indicative of the abundant presence of Sc-MB in the solution. In contrast, Figure S12B demonstrates that, even after multiple washing steps, there is significant retainment of the absorbance in the region. This suggests minimal loss of Sc-MB, underlining the efficiency of the washing steps in retaining the immobilized entities while potentially removing unbound excesses. To further corroborate successful Sc-MB attachment, SERS measurements were conducted. Interestingly, 3 μM Sc-MBs immobilized onto the surface of 20 nm AuNPs exhibited limited SERS enhancement (Figure 4C). While the 3 μM Sc-MBs attached to the 40 nm AuNPs and 80 nm AuNPs resulted in the generation of an intense and comparable SERS signal from the Cy5 modification as shown in Figure 4G,K. Cy5 Raman spectral bands were observed and tentatively assigned as ∼1120 cm^–1^ ν (C–H)ip-bend, ∼1272 cm^–1^ ν (C–N)stretch, ∼1361 cm^–1^ ν(C=C)ring,^24^ and ∼1595 cm^–1^ ν(C=N)stretch modes.^25^Figure S13 illustrates the overall spectra and peak-by-peak comparison as a function of varying the AuNP diameter using a fixed AuNP concentration. Analyzing its peak SERS intensity at its characteristic peaks, as illustrated in Figure S13B–E, compared to the unmodified 40 nm AuNPs, there is a clear increase in SERS signal intensities from 87 to 1250 counts for the peak ∼1565 cm^–1^ as well as from 81 to 1168 counts for the peak ∼1120 cm^–1^ which confirm the presence of Cy5 Raman spectral bands. A decrease in SERS signal was observed as more Sc-MBs were loaded on the surface, which alludes to the dense hairpin packing on the surface of the AuNPs, possibly hindering the reorientation of the Sc-MBs (Figure S14). The surface coverage of Sc-MBs is a critical parameter for SERS detection sensitivity. To further evaluate successful Sc-MB loading, the ζ – potential measurements were obtained (Figure S15), confirming successful attachment due to a decrease in surface charge after Sc-MB modification.

Furthermore, Sc-MB loading on the bare AuNPs was quantified. As illustrated in Figure S16, 57.41, 21.02, and 9.15% of the initial Sc-MB hairpins were successfully immobilized on the bare 20, 40, and 80 nm AuNP surface, respectively. Further confirmation of the successful attachment of Sc-MB to the surface of the AuNPs was confirmed through the hydrodynamic size distribution. An increase from 25.98 ± 1.19 to 45.0 ± 2.86 nm was observed for the 20 nm AuNPs, 46.13 ± 0.78 to 62.67 ± 1.82 nm was observed for the 40 nm AuNPs, and lastly, 86.81 ± 0.87 to 107.56 ± 1.52 nm was observed for the 80 nm AuNPs. The PDI of the Sc-MB functionalized AuNPs confirmed the particles to be highly monodispersed at 0.198 (20 nm), 0.154 (40 nm), and 0.087 (80 nm).

### SERS-Based Hybridization Assay to Confirm a SERS “ON” to “OFF” Configuration

**Figure 5:**
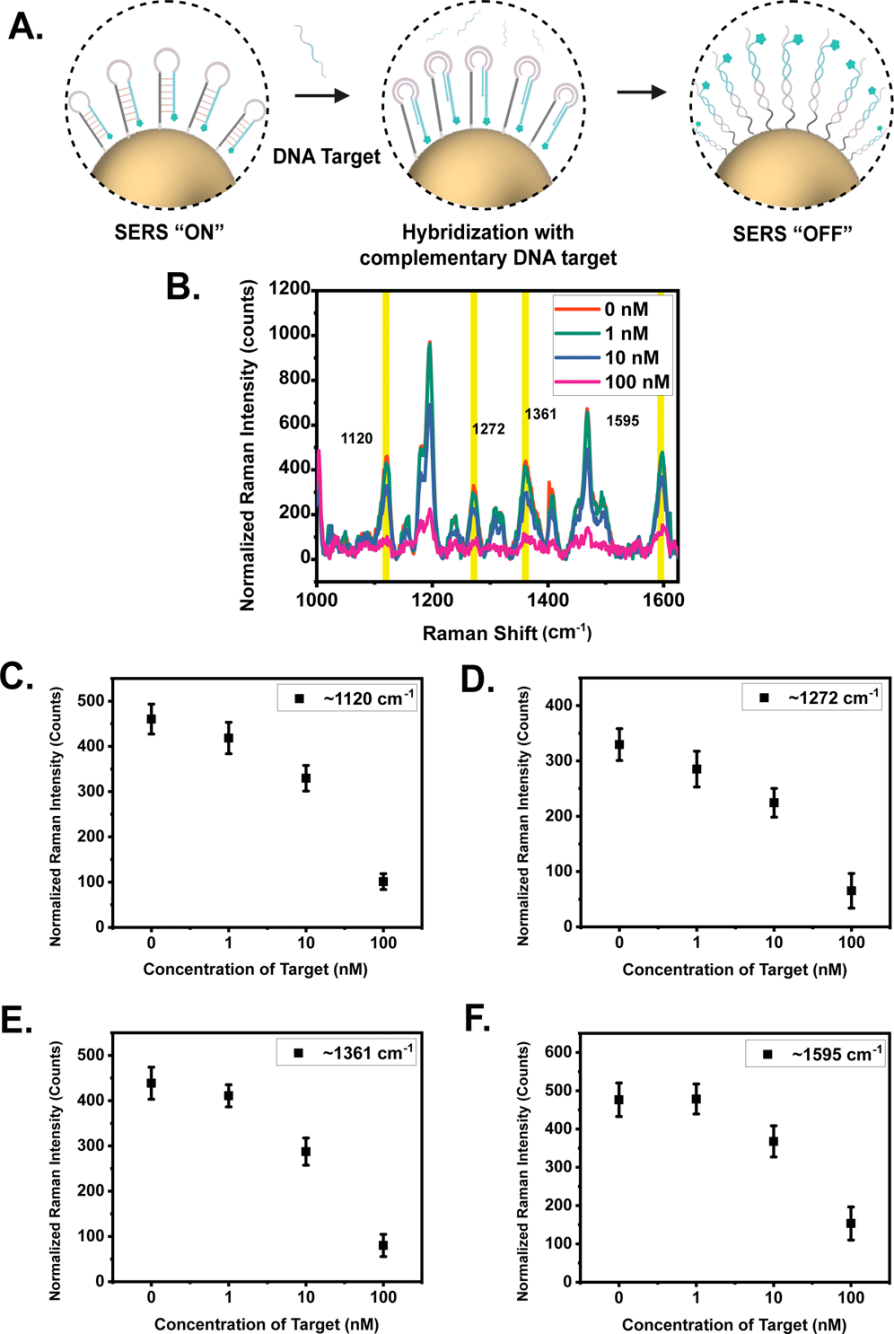
(A) Schematic illustrating a SERS “ON” to “OFF” configuration as a function of increasing target DNA binding occurring due to the displacement of the Cy5 dye away from the AuNP surface. (B) SERS measurement spectra of Sc-MBs functionalized AuNPs in the presence of varying levels of DNA target sequences. (C–F) Analysis of SERS intensity reduction at ∼1120, ∼1272, ∼1361, and 1595 cm^–1^, corresponding to the Cy5 characteristic peaks.

**Figure 6:**
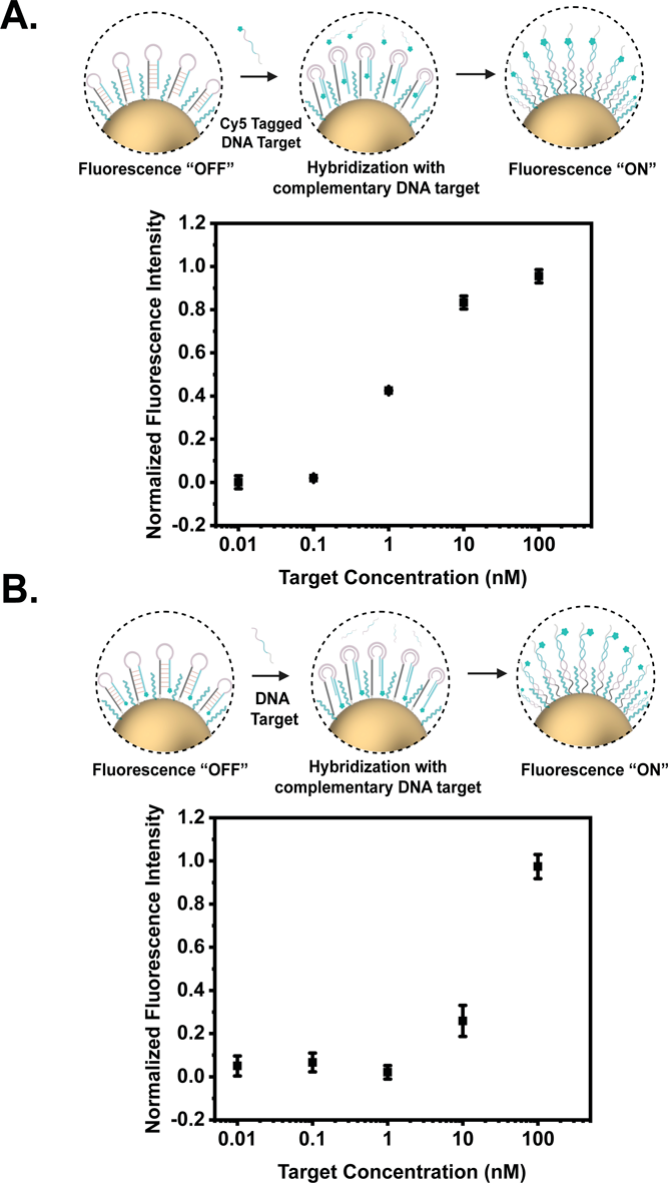
(A) Fluorescence measurements of Sc-HPs–mPEG AuNPs in the presence of varying levels of DNA target sequences conjugated with Cy5. (B) Fluorescence measurement illustrating a Fluorescence “OFF” to “ON” configuration as a function of increasing target DNA binding occurring due to the displacement of the Cy5 dye away from the AuNP surface.

To demonstrate the preservation of the Sc-MB sensing functionality using SERS, quantitative DNA target detection was conducted by incubating Sc-MBs with the target sequence, with various concentrations between 0 to 100 nM. In the absence of the target, high SERS intensity is observed at Cy5 characteristic peaks at ∼1120, ∼1272, ∼1361 and, 1595 cm^–1^ as illustrated in Figure 5B. As expected, upon the introduction of 100 nM of target DNA, as depicted in Figure 5, a marked reduction in SERS intensity was noted, suggesting effective hybridization between the target and the Sc-MBs. This inference was substantiated by the fluorescence quenching data shown in Figure 6. An incremental decline in SERS intensity was observed with target DNA concentrations ranging from 1 to 10 nM, thereby demonstrating a correlation between the increasing presence of target DNA in the reaction mixture and diminishing SERS signal. While the protocol requires further optimization, which is beyond the scope of this work, the current findings suggest that the sensing capabilities of the Sc-MBs remain intact, with the ability to detect DNA targets at concentrations exceeding 10 nM. The overarching trend observed in this research suggests that the freeze-assisted method preserves the hairpin structure during conjugation. This indicates the viability of employing these probes for quantitative analysis in future applications.

### Fluorescence-Based Hybridization Assay to Confirm a Fluorescence “OFF” to “ON” Configuration

To assess the preserved sensing function via fluorescence, we conducted quantitative DNA detection through two distinct studies. The initial study involved hybridization tests with Sc-HPs AuNP complexes postmodification with methoxy(polyethylene glycol) (mPEG), following freeze-assisted immobilization and DNA targets tagged with Cy5. Concurrently, SH-mPEG was coimmobilized on the AuNP surface under freeze-assisted conditions. Despite UV–vis results (referenced in Figure S17) indicating stable AuNP-Sc-MBs-mPEG and AuNP-Sc-HP-mPEG formation, the SERS measurements suggested variable reproducibility, potentially due to inconsistent hairpin and mPEG densities on the AuNP surface, although these data are not depicted. Subsequently, we opted for mPEG immobilization post-freeze, which was proven effective when mPEG’s surface protection was confirmed by NaCl tolerance tests (shown in Figure S18B,C). The AuNP conjugates maintained stability even in 1000 mM NaCl (illustrated in Figure S18C). In a separate fluorescence study to further verify sensing capabilities, Sc-HPs were hybridized with fluorophore-labeled DNA targets. Posthybridization, thorough washing was crucial to remove unbound targets and prevent nonspecific fluorescence.

Analysis of both the supernatant and the AuNP-Sc-HP-mPEG-DNA complex demonstrated a fluorescence intensity rise correlating with increasing DNA target concentration on the AuNPs, as shown in Figures 6A, S19 and S20. The fluorescence intensity of stock solutions and supernatant quantified the amount of DNA targets bound to the Sc-HPs-mPEG-modified AuNPs. In the second study, we used Sc-MBs-mPEG-functionalized AuNPs. Here, nonfluorescent DNA targets were incubated with the conjugates. The ensuing Cy5 displacement from the AuNP surface, triggered by target DNA binding and subsequent hairpin opening, increased fluorescence intensity—a phenomenon observable at concentrations greater than 10 nM due to the quenching effect of the AuNPs on the fluorophore.

## Conclusions

In conclusion, our study represents a significant leap forward in the field of biofunctionalization, particularly in the conjugation of hairpin oligonucleotides to nanoparticles. The innovative freeze-assisted technique that we have refined allows for the efficient and stable attachment of hairpin DNA of varying lengths and molecular beacons to AuNPs. This method preserves the functional integrity and sensing capabilities of the conjugated biomolecules. Utilization of electrophoresis and subsequent SERS and fluorescence measurements robustly confirm the structural and functional retention of these biomolecules postimmobilization.

Notably, the Sc-HPs and Sc-MBs maintain their ability to hybridize with target DNA sequences, thereby underscoring the method’s efficacy in preserving the biorecognition properties of these nucleic acid structures. Moreover, the adaptability of this method to various oligonucleotide structures and its compatibility with different sensing modalities demonstrate its versatility and potential for broad applications. From targeted drug delivery and biosensing to diagnostic platforms, this research paves the way for future innovations in nanobiotechnology and molecular diagnostics. In summary, this research expands the current conventional boundaries of biofunctionalization, offering a novel, rapid, and reliable method for the conjugation of oligonucleotides to nanoparticles.

## Experimental Section

### Chemicals

DNA sequences were purchased from Integrated DNA Technologies. The sequences and their modifications are listed in Table S1. Sodium chloride (NaCl), sodium phosphate monobasic, sodium phosphate dibasic, potassium chloride (KCl), magnesium chloride (MgCl_2_), nuclease-free water (NF water), TCEP, and Tween-20 were purchased from Sigma-Aldrich. Citrate-capped AuNPs (20, 40, 80 nm) were purchased from ABCAM with an OD of 10 (catalog no. ab269936, ab269930, and ab269940 respectively). Nuclease-free water was used for all experiments to prepare the buffers and solutions. Acrylamide/bis-acrylamide solution (29:1), 10× tris-borate-EDTA (TBE buffer), ammonium persulfate (APS), N,N,*N*′,*N*′-tetramethylethylenediamine, SYBR gold stain, and ultralow range DNA ladder (10–300 bp) were all obtained from Invitrogen.

### Instrumentation

The hydrodynamic size (DLS) and the ζ - potential of the AuNPs and snap-cooled DNA hairpin/MBs (Sc-HPs and Sc-MBs) were measured using the Malvern Zetasizer at 25 °C. UV–vis spectrophotometry was performed on a Tecan Infinite 200 Pro microplate across a wavelength range of 350–950 nm. Fluorescence measurements for Cy5 (200 μL per measurement) were obtained using a Qubit 4.0 fluorometer from Fisher Scientific. The loading density of the hairpin oligonucleotides/molecular beacons onto the AuNPs was measured using the supernatant postbioconjugation using a Nanodrop One spectrometer. SERS measurements were performed using a benchtop Raman spectrometer (Wasatch Photonics) equipped with a 785 nm laser. Samples were dispersed into a 384-well greiner black microplate and collected at 8 s integration time, across a wavelength range of 200–2700 cm^–1^. All nanoparticles were measured at a fixed OD of ∼0.9 a.u. All spectra were baseline-corrected prior to analysis using an asymmetric least-squares method applied in MATLAB. BIORAD Mini-Protean system electrophoresis apparatus and BIORAD Gel-doc EZ system were used to run and analyze the Native PAGE gels.

#### Reduction of Disulfide Bond Using TCEP

A 100× molar excess of TCEP was added to the thiolated-DNA sequences to reduce the disulfide bonds. Ten mM of TCEP was prepared in nuclease-free water, of which 25 μL was added to 25 μL of 100 μM of DNA, yielding a total DNA concentration of 50 μM. The solution was then incubated at room temperature in a dark room for 1 h.

#### Snap-Cooling of DNA Hairpin Oligonucleotide (Sc-HPs) and Molecular Beacon (Sc-MBs) Sequences

The DNA hairpins treated with 50 μM of TCEP were heated at 95 °C for 3 min using a heating block, then quickly transferred to ice and allowed to cool there for 20 min.

#### Preparation of Experimental Samples for Gel Electrophoresis

Gel electrophoresis was used to validate the binding of the hairpin sequences to the DNA targets under freeze-assisted conditions. To evaluate this mechanism, the stock hairpin sequences were snap-cooled and then subjected to one freeze–thaw cycle under the same experimental conditions as those with the NPs in the solution. This was achieved by snap-cooling 100 μM Sc-HPs at 95 °C for 3 min and placing them immediately on ice for 20 min. The freeze–thaw cycle condition was completed by adding 10 μL of 100 μM Sc-HPs into a 0.2 mL Eppendorf tube. This was then followed by the addition of 82.1 μL of nuclease-free water, 4.4 μL of 17.6 mg/mL SDS, and 3.5 μL of 1 M NaCl. This reaction mixture now contains a 10 μM Sc-HP stock solution, which is then subjected to one freeze–thaw cycle at −80 °C for 15 min.

For the gel electrophoresis, the experimental reaction mixtures (Lanes 4 and 5, Figure 2) with the target sequence were obtained by mixing 3 μL of 10 μM freeze–thawed hairpin oligonucleotides, 3 μL of 10 μM DNA target sequence, and 3 μL of 1% Tween-20, 21 μL of hybridization buffer 2 were added and vortexed in a 0.200 mL Eppendorf tube. The control samples for this reaction (lane 2, Figure 2) were obtained by mixing 3 μL of 10 μM freeze–thawed hairpin oligonucleotides, 3 μL of nuclease-free water, 3 μL of 1% Tween-20, and 21 μL of hybridization buffer 2 in a 0.200 mL Eppendorf tube. All solutions were incubated in a heating block at 37 °C for 2 h.

Lane 7–10 (Figure 2), which represents the control sample batches, contains Sc-HPs that were not subjected to freeze-assisted conditions. The control samples were obtained by adding 3 μL of 10 μM of snap-cooled hairpin oligonucleotides to 3 μL of nuclease-free water containing 3 μL of 1% Tween-20 and 21 μL of hybridization buffer. Samples containing the target sequence were created by mixing 3 μL of 10 μM of hairpin oligonucleotides with 3 μL of 10 μM of DNA target sequence, 3 μL of 1% Tween-20, and 21 μL of hybridization buffer. All solutions were incubated in a heating block at 37 °C for 2 h.

#### Native PAGE

15% polyacrylamide gels were prepared by mixing an acrylamide/bis-acrylamide solution with 2× TBE (29:1) in equal volumes. To this mixture were added 10% APS and TEMED were added. The gel mixture was dispensed onto the electrophoresis plates and sealed with a comb. This mixture was then allowed to polymerize for 30 min. After, the combs were removed, and the wells were rinsed with nuclease-free water and 1× TBE in successive washes. Samples were then mixed with 20% glycerol and loaded into the wells. The electrophoresis system was run at 15 mA at 4 °C for 3 h to resolve all nucleic acid products completely. The gels are removed and stained with 1× SYBR gold for 30 min, then destained for 30 min. The gels were then visualized to observe the formation of DNA hybrids and species.

#### Freeze-Assisted Immobilization of Sc-HPs and Sc-MBs onto AuNPs

Tables S1 and S2 summarizes the reaction mixtures for Sc-MBs and Sc-HPs as well as the volumes of each component added to 100 μL of AuNPs. Before the DNA hairpins were combined with the AuNPs (20, 40, and 80 nm), 4.4 μL of 17.6 mg/mL SDS was added to 100 μL of AuNP (OD of 1 or 10). The solution was vortexed, and then, 3.5 μL of 1 M NaCl was added while continuously mixing. Different volumes (2, 6, and 12 μL) of 50 μM DNA hairpin oligonucleotides were added to the AuNP suspension and thoroughly mixed. The mixture was subsequently frozen at −80 °C for 15 min. During the thawing process, 10 μL of 1 M NaCl was added to each batch of nanoparticle-DNA suspension to achieve a final NaCl concentration of 100 mM. The suspension was vortexed during thawing to ensure a uniform mixing of NaCl with the AuNP solution. It was crucial to ensure the mixing of the solution during the thawing phase. Following this, the mixture was incubated overnight in the dark. The DNA hairpin-AuNPs were then centrifuged according to the speeds and times specified in Table S3. The supernatant was collected to help quantify the DNA loading on the AuNPs. At the same time, the Au-nanoprobes were washed with hybridization buffer (150 mM NaCl, 100 mM phosphate buffer, 12 mM MgCl2, 10 mM KCl, and 0.1% Tween-20) before they were resuspended in 100 μL of the same buffer. To test the stability of the Au-nanoprobes, 50 μL of Au-nanoprobes (OD 1) were suspended in 50 μL of nuclease-free water, and aliquots of a 5 M NaCl solution were added to the Au-nanoprobes so that the final salt concentration ranged from 0 to 1000 mM NaCl, and the samples were measured using UV–vis.

#### SERS-Based Hybridization Assay Utilizing the Sc-MBs – AuNPs

Eighteen μL of AuNP–MBs (OD ∼ 5.5) in hybridization buffer 1 (Table S4) was combined with 2 μL of DNA target present at 4 different concentrations; 0, 10, 100, and 1000 nM). The suspension was allowed to incubate for 2 h at room temperature. Subsequently, 20 μL of the solution was transferred to a 384-well microplate for the measurement.

#### PEGylation of AuNP – Sc-MBs and AuNP – Sc-HPs for Fluorescence Measurements

After the freeze-assisted conjugation and washing steps, AuNPs with attached MBs were suspended in 100 μL of nuclease-free water by adding 10 μL of 9 μM SH-mPEG_2000_ (*M*_w_: 2000 Da). After being allowed to incubate for 30 min, the particles were centrifuged and washed twice using hybridization buffer.

#### Fluorescence-Based Hybridization Assay Utilizing Sc-HPs and Cy5-Tagged Target DNA Sequence for Direct Target Sequence Quantification

In order to directly quantify the target sequences hybridized with the Sc-HPs, the target sequences were modified with a Cy5 dye instead of the hairpin probes. To quantify target-binding, this was achieved by measuring the fluorescence of the AuNP-DNA hairpin–hybridized with the target sequence pellet as well as the supernatant, which contains the excess DNA target sequences. For this study, 21 μL portion of AuNP conjugated with mPEG and DNA hairpin oligonucleotides (∼ OD 5.5) in hybridization buffer 2 was added to a 0.2 mL PCR tube. This was then followed by the addition of 168 μL of hybridization buffer 2 (Table S4) and 21 μL of the respective stock DNA target with *x* nM (*x* = 0, 1, 10, 100, 1000 nM). The mixture was then vortexed and transferred to a heating block. At 37 °C, the 0.2 mL PCR tubes with the reaction mixture were then incubated for 2 h. The reaction mixture was then transferred to a 1.5 mL Eppendorf tube and centrifuged at the speed illustrated in Table S3 for the respective AuNP size used. The samples were then washed in hybridization buffer 2–3 times and resuspended in 210 μL of hybridization buffer 2. The pellet and the supernatant were quantified using a fluorometer. For measurements, 200 μL of the reaction mixture was then transferred to the Quibit 4.0 Assay tubes, and each concentration was measured in triplicate with >30 s in between measurements.

#### Fluorescence-Based Hybridization Assay Utilizing Sc-MBs (Cy5-Tagged) and Target DNA Sequence for an Indirect Target Sequence Quantification

Twenty-one μL of AuNP conjugated with mPEG and DNA hairpin oligonucleotides (∼ OD 5.5) in hybridization buffer 2 was added to a 0.2 mL PCR tube. This was then followed by the addition of 168 μL of hybridization buffer 2 and 21 μL of respective stock DNA target with *x* nM (*x* = 0, 1, 10, 100, and 1000 nM). The mixture was then vortexed and transferred to a heating block. At 37 °C, the 0.2 mL PCR tubes with the reaction mixture were then incubated for 2 h. Without washing, 200 μL of the reaction mixture was then transferred to the Quibit 4.0 Assay tubes, and each concentration was measured in triplicate with >30 s in between measurements.

## Supporting Information Available

Characterization of commercially bought AuNPs, NUPACK simulation of designed hairpin oligonucleotides, characterization of freeze-assisted protocol non-TCEP treated, information regarding the probe density loaded on the AuNPs, conjugation and characterization of hairpin oligonucleotides of longer sequences, stability of hairpin-AuNP conjugates under high salt concentration, effect of high surface density packing on SERS measurements, fluorescence measurement standard curves and raw fluorescence intensity measurements (PDF)

## Abbreviations

AuNP: gold nanoparticles
Sc-HPs: snap-cooled hairpin oligonucleotides
Sc-MBs: snap-cooled hairpin molecular beacons
SERS: surface-enhanced Raman spectroscopy
Cy5: Cyanine 5
nt: nucleotides
bp: base pairs
PEG: poly ethylene glycol
TCEP: tris(2-carboxyethyl)phosphine
DTT: dithiothreitol
miR-574–5p: micro-RNA-574–5p
SDS: sodium dodecyl sulfate
