# Supplementary material for: Freeze-Driven Synthesis of DNA Hairpin-Conjugated Gold Nanoparticle Biosensors for Dual-Mode Detection
Source: ACS Appl Bio Mater. 2024 Apr 17;7(5):3005–13. doi: 10.1021/acsabm.4c00069 (PMC11110043; doi:10.1021/acsabm.4c00069)
Supplement: Supplementary file 1 — mt4c00069_si_001.pdf [file mt4c00069_si_001.pdf]

Supporting Information

Freeze-Driven Synthesis of DNA Hairpin-Conjugated Gold Nanoparticle Biosensors for Dual-Mode Detection

Angela Michelle San Juan[1,2], Siddhant Jaitpal[1], Ka Wai Ng[1], Cecilia Martinez[1], Sayantan Tripathy[1,2], Christian Phillips[1], Gerard Cote[1,2], Samuel Mabbott[1,2,*]

[1]Department of Biomedical Engineering, Texas A&M University, College Station, TX

[2]Center for Remote Health Technologies & Systems, Texas A&M Engineering Experiment Station, College Station, TX

Email: smabbott@tamu.edu*

Table of Contents

1. Freeze-Assisted Immobilization of Sc-HPs and Sc-MBs onto bare AuNPs 5

2. Characterization of Commercially Bought bare AuNPs (20 nm, 40 nm, 80nm) 7

3. DNA Hairpin Sequences and NUPACK Simulation 8

4. NUPACK Simulation of Sc-HPs binding with DNA Target at 37 ^{o}C 10

5. Non-TCEP Treated Hairpin Oligonucleotide Functionalization onto bare AuNP 11

6. Improved Stability of bare AuNPs using SDS tested with varying concentration of NaCl 12

7. Stability of AuNP immobilized Sc-HPs and Sc-MBs under varying Salt Conditions (OD1) Sc-HP 6 uM, Sc-MB 3 uM (n=5) 13

8. Sc-HPs Oligonucleotide Loading Quantification using a Nanodrop Spectrophotometer (AuNP OD 10 – Freeze Assisted) 15

9. Freeze – Assisted Protocol immobilizing Sc-HPs using longer sequences and varying bare AuNP diameters 16

10. Freeze – Assisted Immobilization of Sc-HP$_2$ (55 nt long) onto bare AuNP 18

11. Freeze – Assisted Immobilization of Sc-HP$_3$ (70 nt long) onto bare AuNPs 19

12. Dynamic Light Scattering Measurements of Freeze-Assisted immobilized Sc-HP$_2$ and Sc-HP$_3$ - AuNPs 20

13. ζ-Potential Measurements of Freeze-Assisted immobilized Sc-HP$_2$ and Sc-HP$_3$ - AuNPs 21

14. UV-Vis Measurement of 40 nm with AuNP (OD 1) – Sc-MBs before and after centrifugation ... 22

15. SERS Measurement of 20 nm, 40nm, and 80 nm AuNP – Sc-MBs (3 µM) 23

16. SERS Measurement as a function of initial Sc-MBs added in the reaction illustrating effects of packing densities (OD ~2.5) 25

17. ζ-Potential Measurements of 20 nm, 40nm, and 80 nm AuNP – Sc-MBs (3 µM) 25

18. Sc - MBs Loading Quantification using a Nanodrop Spectrophotometer 27

19. Characterization of Sc-MBs co-immobilization with SH-mPEG using the modified freeze-assisted conditions. 28

20. Characterization of Sc-HP-mPEG post freeze-assisted immobilization via NaCl tolerance test. 29

21. Cy5 tagged DNA Target Standard Curve and Supernatant Samples 30

22. Cy5 tagged DNA Target Standard Curve vs Supernatant of Immobilized DNA Target to 40 nm AuNP – Hairpin Oligos (3 µM) 31

23. Raw Fluorescence Intensity of washed 40 nm AuNP – Hairpin Oligos (3 µM) 32

1. Freeze-Assisted Immobilization of Sc-HPs and Sc-MBs onto bare AuNPs

Volumes added to immobilize Sc-MBs (Molecular Beacons) ontu AuNPs

Table S1. Illustrates the of SDS, NaCl, and oligonucleotide volume to 100 µL of AuNPs solution with the corresponding optical density purchased from the manufacturer before the freeze-assisted immobilization process.

Diameter	*Optical Density*	*17.6 mg/mL SDS (µL)*	*Initial 1 M NaCl (µL)*	*Oligonucleotide*	*Oligonucleotide Concentration (µM)*	*Volume of Oligonucleotide (µL)*	*1 M NaCl during thawing (µL)*
20 nm	*10*	*13.2*	*3.5*	*MBs*	*3*	*6*	*10*
40 nm	*10*	*4.4*	*3.5*	*MBs*	*3*	*6*	*10*
80 nm	*10*	*4.4*	*3.5*	*MBs*	*3*	*6*	*10*

Volumes added to immobilize Sc-HPs (Hairpin Oligonucleotides) ontu AuNPs

Table S2. Illustrates the of SDS, NaCl, and oligonucleotide volume to 100 µL of AuNPs solution with the corresponding optical density purchased from the manufacturer before the freeze-assisted immobilization process.(*Optimized formulation used for SERS based and Fluorescence based assay)*

Diameter	*Optical Density*	*17.6 mg/mL SDS (µL)*	*Initial 1 M NaCl (µL)*	*Oligonucleotide*	*Oligonucleotide Concentration (µM)*	*Volume of Oligonucleotide (µL)*	*1 M NaCl during thawing (µL)*
40 nm	*1 & 10*	*4.4*	*3.5*	*Hairpin Oligos*	*3*	*6*	*10*

Centrifugation Speeds

Table S3. Illustrates centrifugal speeds and duration used in order to pellet down AuNPs of varying size ranging from 20, 40, and 80 nm AuNPs

Diameter	*Centrifugation Speed (rpm)*	*Duration (min)*
20 nm – MBs	*10,000*	*5*
40 nm – MBs / Hairpin Oligos	*5,000*	*5*

80 nm - MBs	*5,000*	*1*

Buffers

Table S4. Illustrates hybridization buffer components for SERS based and Fluorescence based assays.

Hybridization Buffer 1	*150 mM NaCl, 100 mM Phosphate Buffer, 12 mM MgCl2, 10 mM KCl*
Hybridization Buffer 2	*150 mM NaCl, 100 mM Phosphate Buffer, 12 mM MgCl2, 10 mM KCl, 0.1% Tween 20*

2. Characterization of Commercially Bought bare AuNPs (20 nm, 40 nm, 80nm)

Table S5. illustrates the average sizes and the standard deviation based on the number % value of the commercially acquired AuNPs.

Diameter	*λmax*	*AuNP Optical Density (A.U.)*	*Z-average Hydrodynamic Diameter (d.nm)*	*PDI*
20 nm	*520 nm*	*10*	*25.96 ± 1.76*	*0.161 ± 0.05*
40 nm	*525 nm*	*10*	*37.93 ± 1.57*	*0.163 ± 0.01*
80 nm	*540 nm*	*10*	*78.55 ± 3.91*	*0.076 ± 0.02*

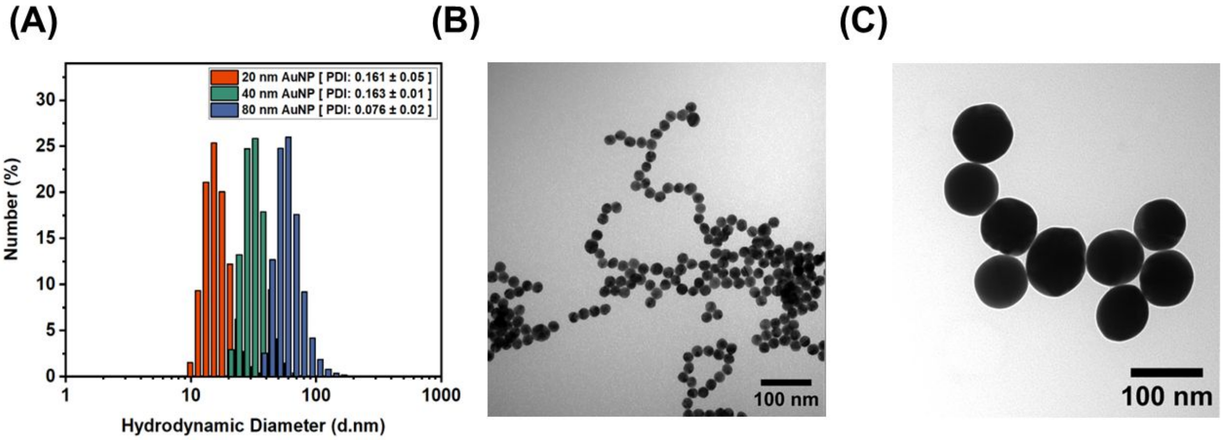

Figure S1. Illustrates the (A) hydrodynamic diameter, (B-C) TEM images, and polydispersity index of the commercially bought AuNPs. Hydrodynamic size measurements were conducted on the commercially acquired AuNPs from ABCAM. These samples exhibited a small polydispersity index (PDI) and offered an excellent substrate to evaluate the modified freeze-assisted protocol.

3. DNA Hairpin Sequences and NUPACK Simulation

Table S6. illustrates the sequences of the hairpin simulated via NuPACK, DNA target sequence, and lastly, hairpin molecular beacons containing 5' dithiol group and 3' Cy5 group.

DNA Sequence Name	Sequence 5' → 3'	Nucleotide Length
DNA Target	5'TGAGTGTGTGTGTGTGAGTGTGT 3'	23
Sc-HPs 5' Thiol	5' Dithiol – CGGTACACACACTCACACACACACACTCAGTACCG 3'	35
Sc-MBs 5' Thiol-Cy5-3'	5' Dithiol – CGGTACACACACTCACACACACACACTCAGTACCG – Cy5 3'	35

Table S7. Illustrates the DNA hairpin and molecular beacon sequences with their corresponding molecular weights and nanodrop concentrations after TCEP reduction. Additionally, it also illustrates the sequence and molecular weight of the DNA target—analogue of miR-574-5p.

	DNA Sequence Name	Molecular Weight	Nucleotide Length (nt)	Molarity	Nanodrop Concentration (ng/ µL)
1	Sc-HPs 5' Thiol	10,782.1	35	48.22 µM	521.09 ± 2.9
2	Sc-MBs 5' Thiol-Cy5-3'	11,452.8	35	53.689 µM	615.9 ± 1.9
3	DNA Target	7,227.7	23		

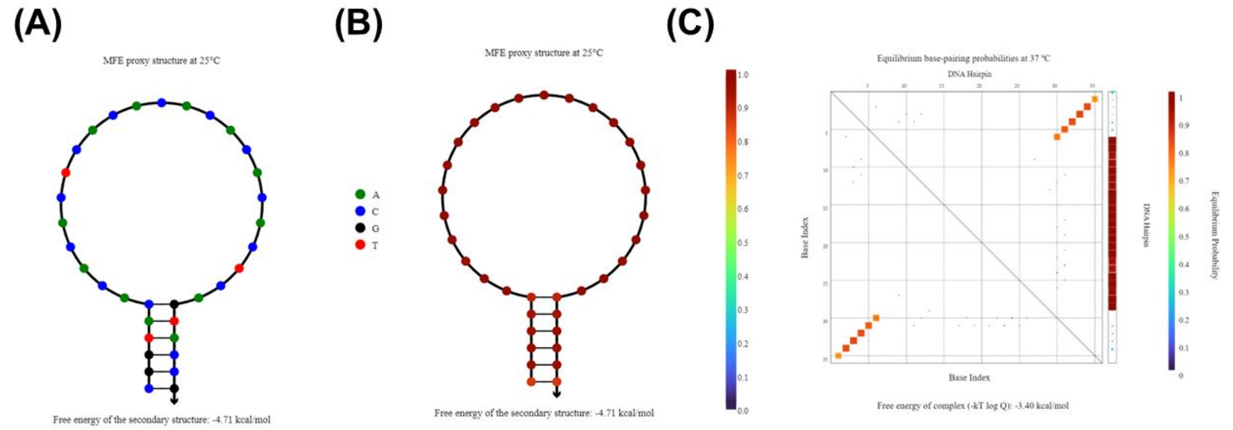

Figure S2. (A-B) Illustrates the secondary structures of the DNA hairpin sequences simulated using NuPACK. (C) Depicts the equilibrium base pairing probabilities of the simulated DNA hairpin at 37 ^{o}C. NuPACK allows for analysis of the DNA hairpins designed, including the equilibrium probabilities of different secondary structures. In summary, at 25 °C with 1 M NaCl, the modeled hairpin sequences showed a high likelihood that base pairing would occur within the stem structures and a loop region would be formed.

4. NUPACK Simulation of Sc-HPs binding with DNA Target at 37 °C

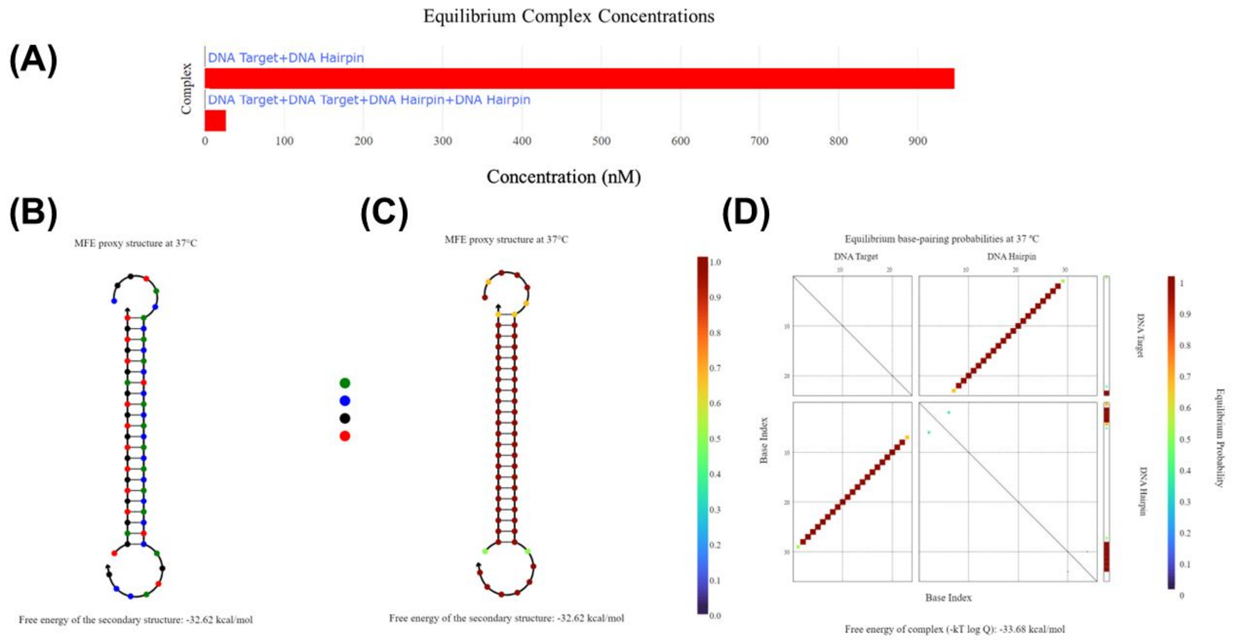

Figure S3. (A) Depicts the proportion of products formed within this reaction mixture once conducted at 37 °C. (B-D) Illustrates the DNA hairpin and DNA target sequence binding simulated via NuPACK. NuPACK simulation of the binding event between the target sequence and designed hairpins illustrates a thermodynamically stable product formation with a ΔG of -32.62 kcal/mol.

5. Non-TCEP Treated Hairpin Oligonucleotide Functionalization onto bare AuNP

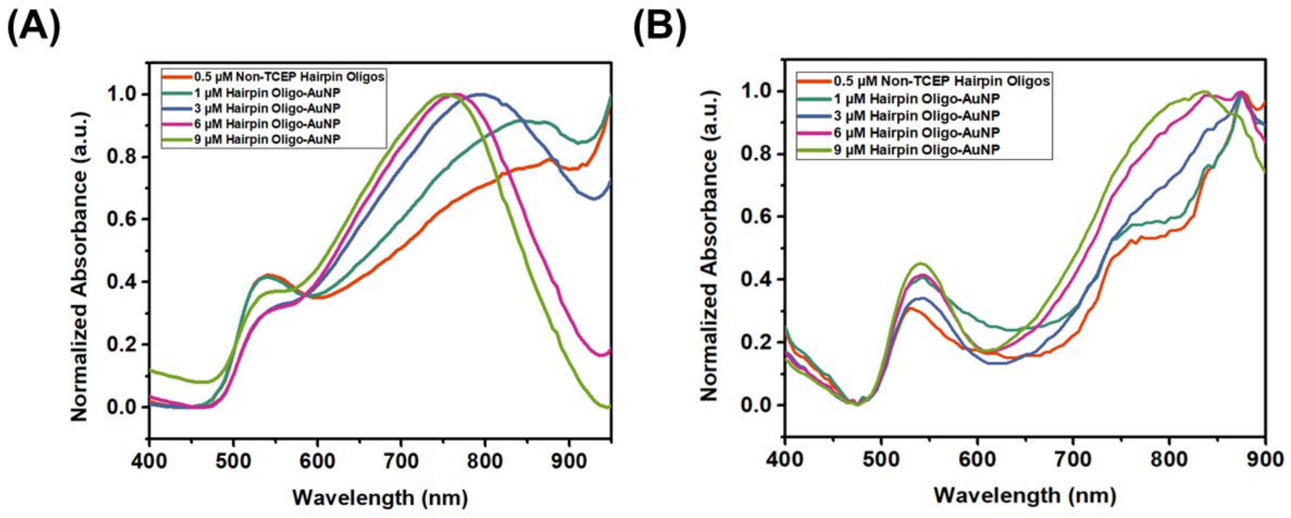

Figure S4. Utilizing (A) 20 nm and (B) 40 nm AuNPs with OD 10 (n=3), freeze assisted oligonucleotide adsorption as reported by Liu et. al[1] was employed with varying initial concentration of non-TCEP treated DNA hairpin oligonucleotides. (DNA hairpins were snap cooled prior to loading with AuNPs).

6. Improved Stability of bare AuNPs using SDS tested with varying concentration of NaCl

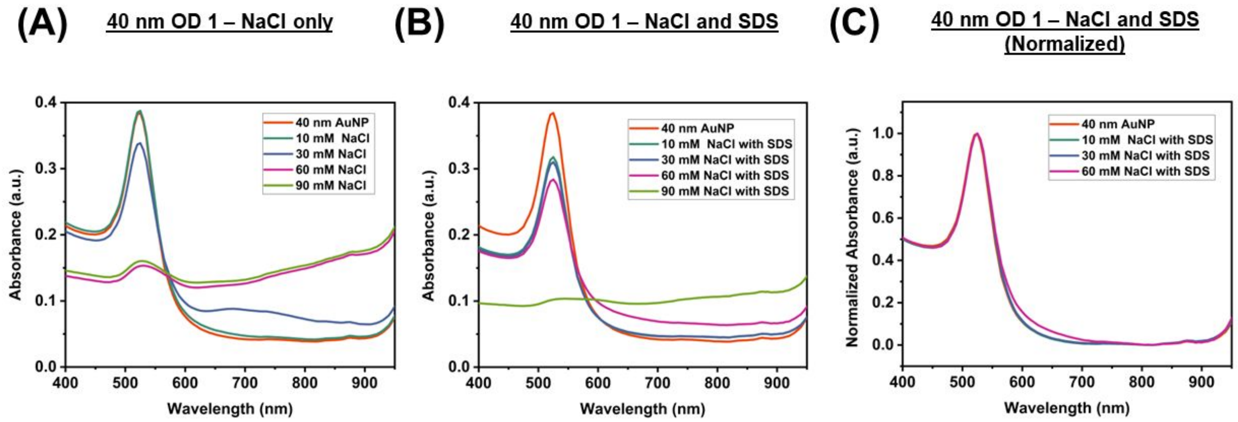

Figure S5. (A) UV-Vis spectra of 40 nm AuNP with an OD of 1 with varying concentrations of NaCl illustrating aggregation of nanoparticles at > 60 mM NaCl. 100 µL of AuNP OD 10 was added with 1 µL, 3 µL, 6 µL, and 9 µL of 1 M NaCl (n=3). (B) UV-Vis spectra of 40 nm AuNP with an OD of 1 with varying concentration of NaCl and fixed SDS concentration illustrating improved AuNP stability. (C) Normalized UV-Vis spectra showing stability < 60 mM NaCl concentration.

7. Stability of AuNP immobilized Sc-HPs and Sc-MBs under varying Salt Conditions (OD1) Sc-HP 6 uM, Sc-MB 3 uM (n=5)

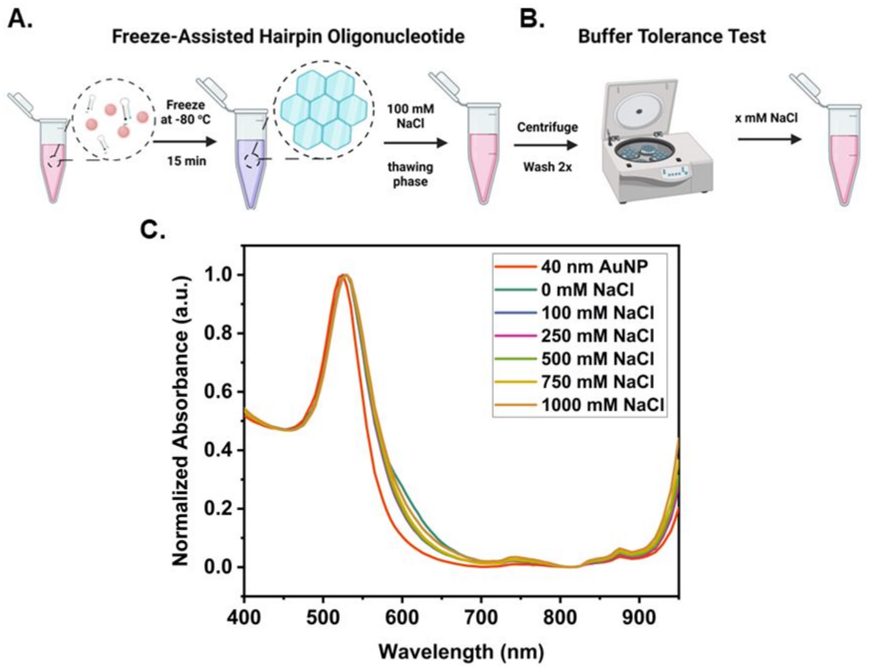

Figure S6. For this experiment, 50 µL of hairpin oligonucleotide-AuNP (OD 1) were added with 50 µL of nuclease-free water, yielding an OD of ~0.5. UV-Vis measurements were taken at 0 mM, 100 mM, 250 mM, 500 mM, 750 mM, and 1000 mM NaCl concentrations by incrementally adding 5 M NaCl into the sample. This was achieved by adding the following volumes of 5 M NaCl, as illustrated in the table below. Each experiment was repeated five times. (A-B) depicts the methodology employed for the NaCl buffer tolerance test, spiking the 6 µM Sc-HP AuNP conjugates with varying levels of NaCl from 0 to 1000 mM NaCl. (C) UV-vis measurements illustrate the stability of Sc-HP-functionalized AuNPs at a high salt.

Table S8. Depicts the incremental volume addition of 1 M NaCl to each well to evaluate the stability of the AuNP-DNA hairpin conjugates.

**NaCl Concentration**	**5 M NaCl – Total volume spiked (µL)**	**5 M NaCl – Total volume spiked per measurement on the same well (µL)**	**Volume of AuNP with an OD of ~0.5 (µL)**
0	0	0	100
100 mM	2.05	+ 2.05	100
250 mM	5.28	+ 3.23	100
500 mM	11.125	+ 5.845	100
750 mM	17.65	+ 6.52	100
1000 mM	25	+ 7.35	100

8. Sc-HPs Oligonucleotide Loading Quantification using a Nanodrop Spectrophotometer (AuNP OD 10 – Freeze Assisted)

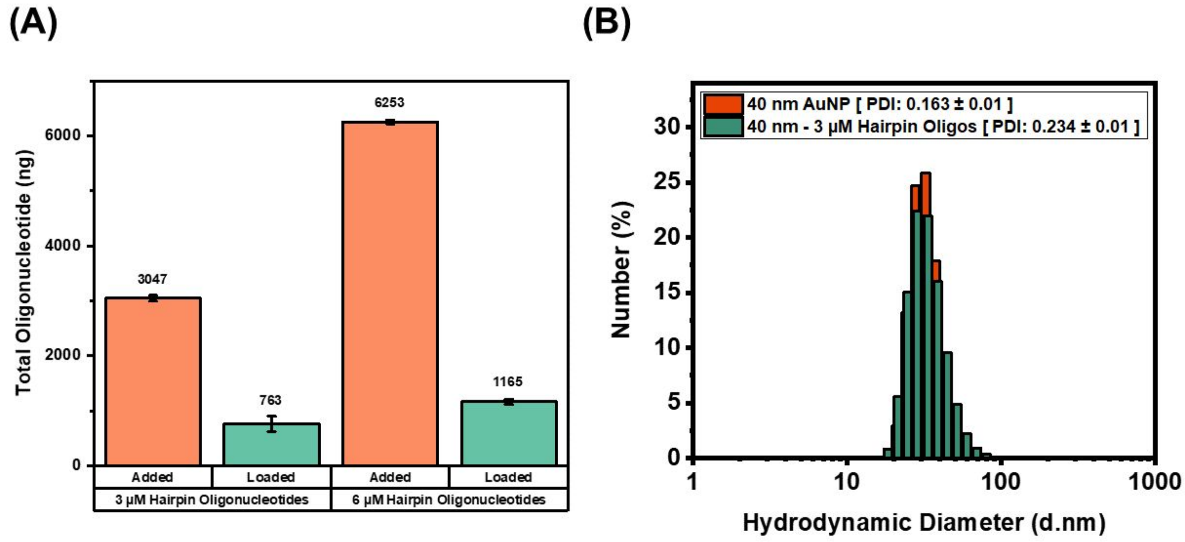

Figure S7. To further validate that the DNA hairpin was adsorbed on the surface, the stock of the DNA hairpin and the supernatant obtained from the reaction were measured on the nanodrop. By calculating the total mass of oligonucleotides added to the solution and the total mass of oligonucleotides in the supernatant, the number of oligonucleotides loaded on the surface was obtained. A further increase in loading was seen as the initial concentration of DNA hairpins was added in the process. For 3 μM of initial DNA hairpins added, ~25% were successfully loaded. While 6 μM of initial DNA hairpins were added, 18.6% were successfully loaded. Each experiment was repeated three times. (A) Nanodrop measurements of oligonucleotide added to the 100 μL AuNP solution with SDS and NaCl before freezing and the amount loaded after Sc-HPs immobilization (washed twice). (B) Dynamic Light scattering measurements of 40 nm AuNP (OD 10) and Sc-HPs immobilized AuNPs.

9. Freeze – Assisted Protocol immobilizing Sc-HPs using longer sequences and varying bare AuNP diameters

Table S9. Illustrates the DNA hairpin and with longer sequences of 55 nt and 70 nt respectively as well as its corresponding molecular weight.

**Hairpin Name**	**Nucleotide Length**	**Nucleotide Sequence 5' → 3'**	**Molecular Weight (g/mol)**
Sc-HP_2	55	5' Dithiol – Sp18- GTTCTCTCGAGACAAGGGAGTGATGAC ACTCCACCATGAATCACTCCCTTGTCTC -3'	17,035
Sc-HP_3	70	5' Dithiol – Sp18- TCATGGTGGAGTGTCTTCATCAC GTTCTCTCATCACTCCCTTGTCTCGAGAGA ACGTGATGAAACCATGA - 3'	22, 039

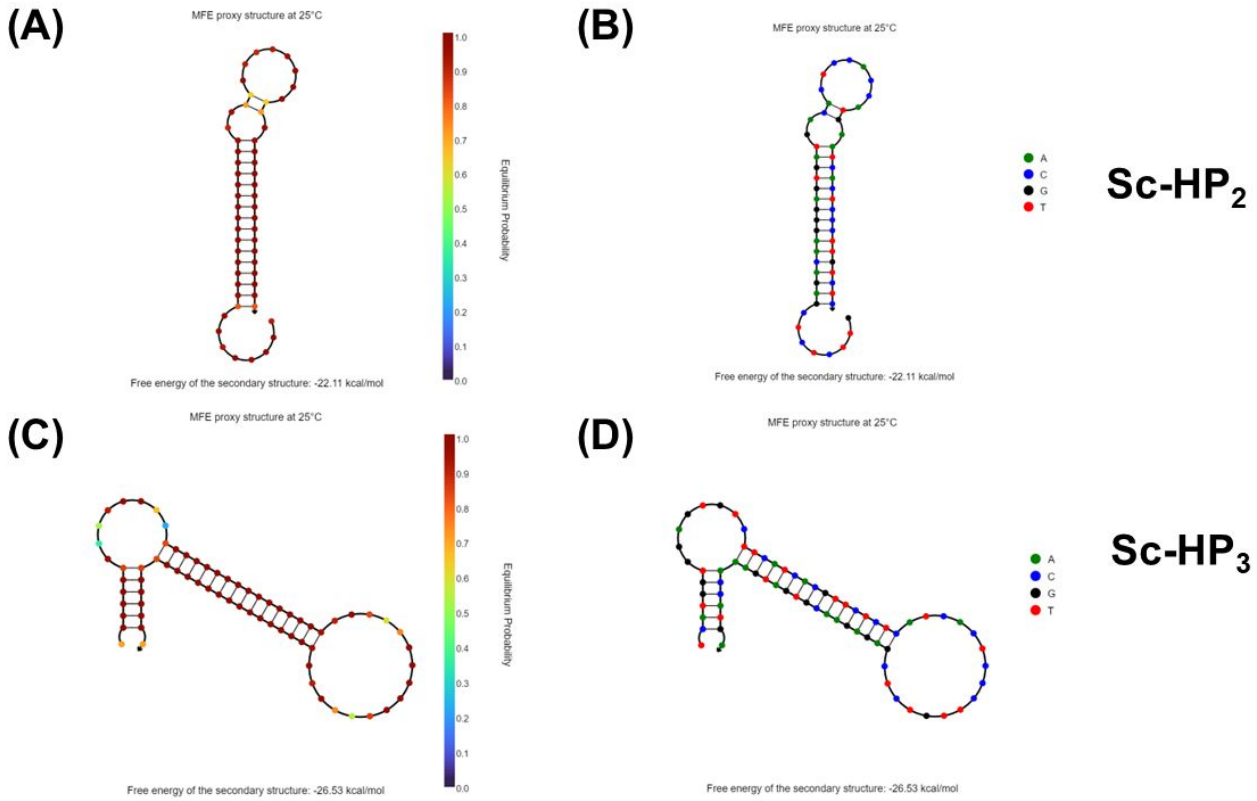

Figure S8. Illustrates the secondary structures of the DNA hairpin sequences (55 nt and 70 nt long) simulated using NuPACK. NuPACK allows for analysis of the DNA hairpins designed, including the equilibrium probabilities of different secondary structures. In summary, at 25 °C with 1 M NaCl, the modeled hairpin sequences showed a high likelihood that base pairing would occur within the stem structures and a loop region would be formed. (A-B) represents the simulated secondary structure of the 55-nt snap-cooled hairpin sequence (Sc-HP$_2$) and (C-D) represents the simulated secondary structure of the 70-nt snap-cooled hairpin sequence (Sc-HP$_3$).

10. Freeze – Assisted Immobilization of Sc-HP_2 (55 nt long) onto bare AuNP

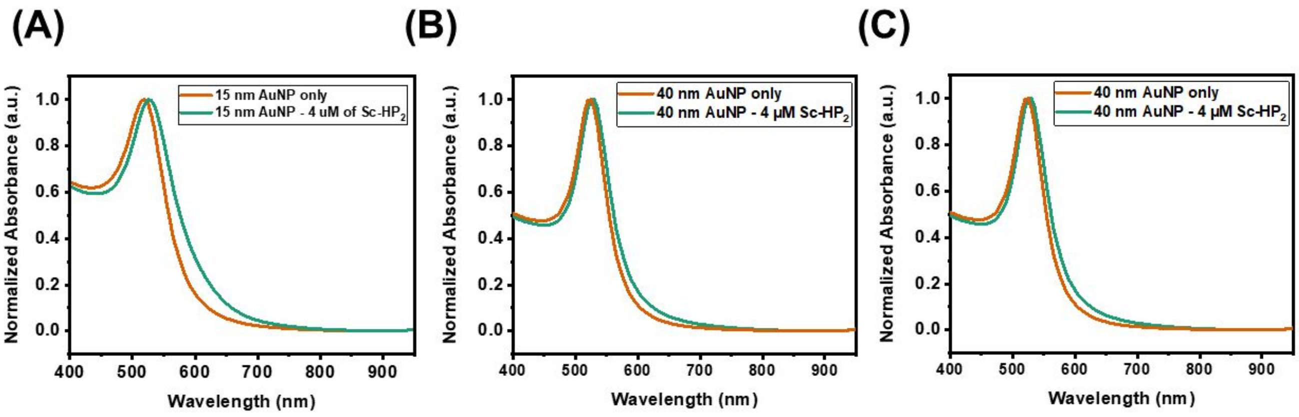

Figure S9. In this experiment, 100 µL AuNP (OD 10) was incubated with 13.2 µL of 17.6 mg/mL SDS, and 12.5 µL of 32 µM TCEP treated Sc-HP_2 without any NaCl addition. The samples were vortexed after addition of each reagent and frozen at -80 ^{o}C for 15 minutes, followed by the addition of 10 µL of 1 M NaCl during the thawing phase. As the AuNP size diameter increased to 60 nm AuNP, it was observed that it is more sensitive to aggregation with the addition of NaCl. To achieve stable biofunctionalization, the optimized conditions were explored without any NaCl addition pre and post freeze-assisted conjugation. Each experiment was repeated three times. (A-B) Depicts UV-Vis measurements of 15 nm and 40 nm AuNP (OD 10) and AuNP functionalized with an initial 4 µM of Sc-HP2 (54 nt long). The freeze assisted conjugation method was achieved without any initial NaCl addition and 100 mM NaCl during the thawing phase. In this study, Sc-HP_2 stocks had a final concentration of 32 µM TCEP treated. (C) Depicts a UV-Vis measurement of 60 nm AuNP (OD 10) and AuNP functionalized with an initial 4 µM of Sc-HP_2 (54 nt long). For attachment of the hairpins to the 60 nm AuNPs, no NaCl was required pre or post freeze-assisted labelling.

11. Freeze – Assisted Immobilization of Sc-HP$_3$ (70 nt long) onto bare AuNPs

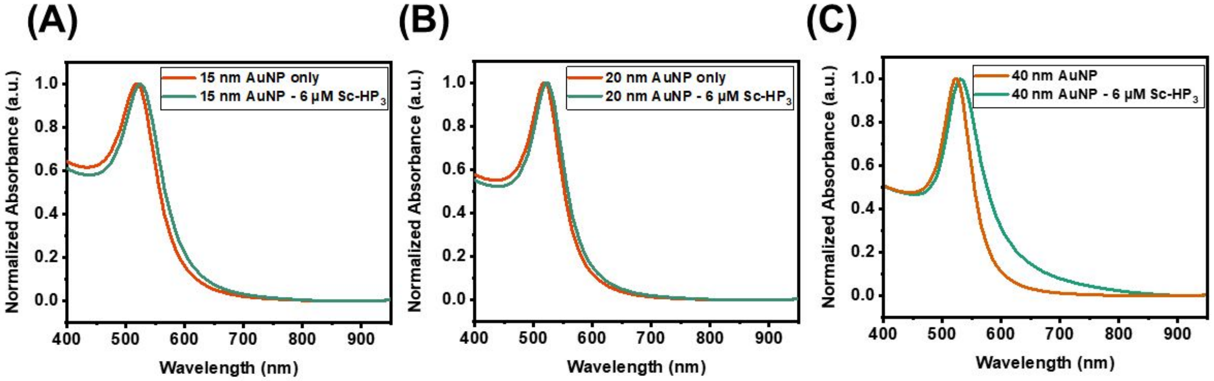

Figure S10. (A-B) Depicts a UV-Vis measurement of 15 nm and 20 nm (OD 10) AuNP functionalized with an initial 6 µM of Sc-HP$_3$ (70 nt long). This process was achieved by incubating 100 µL AuNP (OD 10) followed by the addition of 13.2 µL of 17.6 mg/mL SDS and 12 µL of 50 µM TCEP treated Sc-HP$_3$. The samples were vortexed at each sequential addition of the reagents. The samples were frozen for 15 minutes followed by the addition of 10 µL of 1 M NaCl during the thawing process. (C) UV-Vis measurement of 40 nm AuNP and AuNP functionalized with an initial 6 µM of Sc-HP2 (70 nt long). Spectral broadening is seen after DNA functionalization. This could be further improved by increasing the concentration of the initial oligonucleotides added as well as exploring the role of NaCl addition post-functionalization during the thawing phase. It was seen that no NaCl was best for 60 nm AuNPs pre or post functionalization. Therefore, it could be hypothesized the same conditions could be applied to improve biofunctionalization during the process as the oligonucleotide sequences are longer. Each experiment was repeated 3 times.

12. Dynamic Light Scattering Measurements of Freeze-Assisted immobilized Sc-HP_2 and Sc-HP_3 - AuNPs

Table S10. The table shows the hydrodynamic diameter and polydispersity index of each AuNP and AuNP – Sc-HP conjugates with varying lengths of 54 nt and 70 nt long. Each measurement was repeated 3 times.

Sample Name	Number (%)	PDI
15 nm AuNP	10.89 ± 0.44	0.149 ± 0.09
15 nm AuNP – Sc-HP_2	32.94 ± 3.15	0.317 ± 0.069
15 nm AuNP – Sc-HP_3	27.02 ± 3.18	0.336 ± 0.058
20 nm AuNP	18.22 ± 0.36	0.182 ± 0.019
20 nm AuNP – Sc-HP_3	38.78 ± 6.52	0.270 ± 0.052
40 nm AuNP	28.52 ± 3.38	0.163 ± 0.05
40 nm AuNP – Sc-HP_2	48.55 ± 4.25	0.147 ± 0.025
40 nm AuNP – Sc-HP_3	78.21 ± 13.026	0.215 ± 0.041
60 nm AuNP	42.41 ± 3.44	0.234 ± 0.01
60 nm AuNP – Sc – HP_2	65.78 ± 1.71	0.090 ± 0.012

13. ζ-Potential Measurements of Freeze-Assisted immobilized Sc-HP_2 and Sc-HP_3 - AuNPs

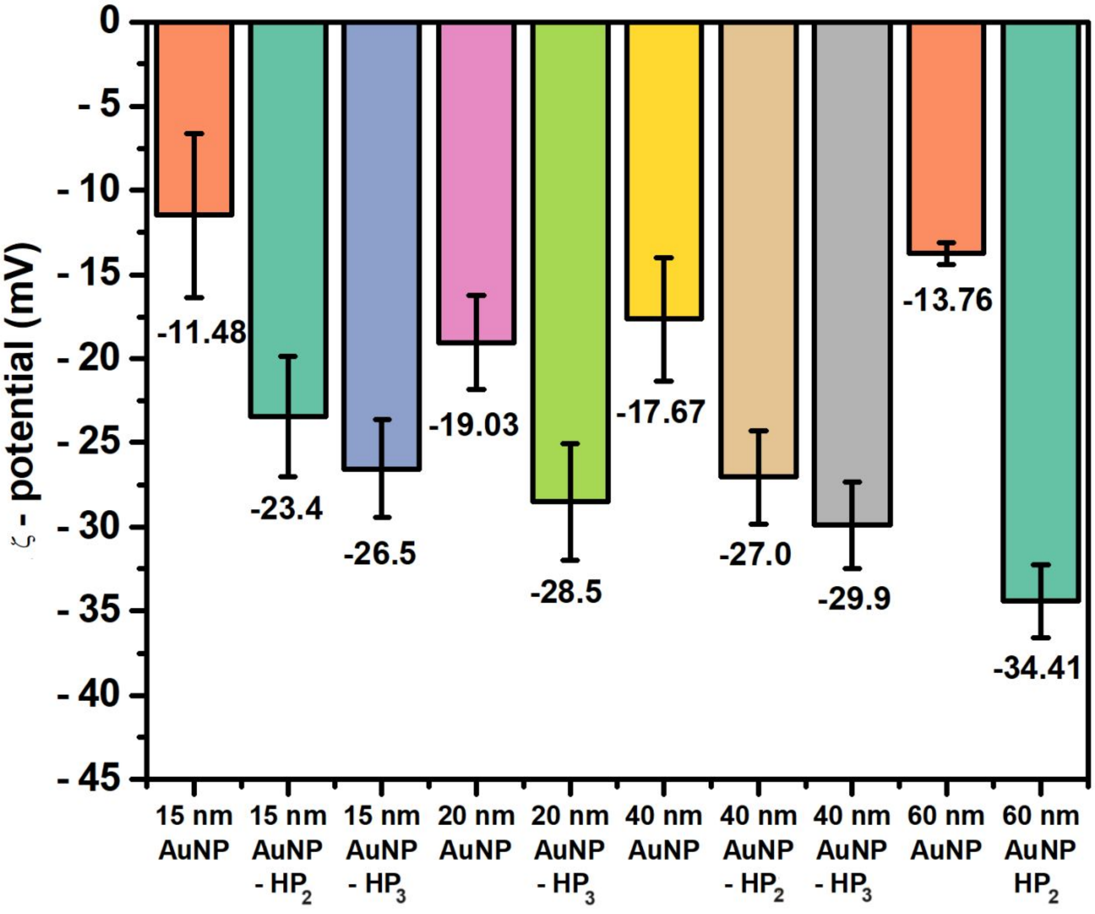

Figure S11. ζ- potential measurements of 15, 20, and 60 nm AuNP before and after Sc-HPs immobilization on the AuNP surface. Each measurement was repeated 3 times.

14. UV-Vis Measurement of 40 nm with AuNP (OD 1) – Sc-MBs before and after centrifugation

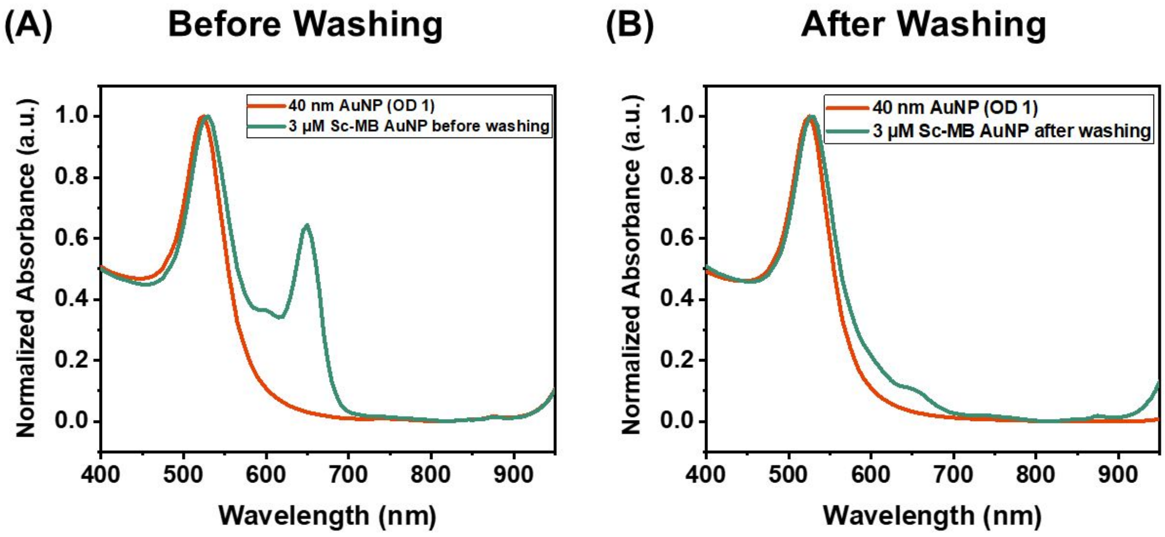

Figure S12. The UV-Vis spectra represent AuNP-DNA hairpin-Cy5 conjugates after the freeze-assisted immobilization, with a peak observed at ~650 nm corresponding to the absorbance of the Cy5 dye. B illustrates the overall spectra of the AuNP-DNA hairpin-Cy5 conjugates after several washing steps. The retention of the small peak at ~650 nm is attributed to the DNA-Cy5 immobilized on the surface of the AuNPs. Each measurement was repeated 3 times. (A) Demonstrates UV-Vis spectra before and after washing of the freeze-assisted AuNPs after conjugation. Illustrates the absorbance of the excess Cy5 dye. (B) UV-Vis spectra of 40 nm AuNP with an OD of 1 with varying concentration of NaCl and fixed SDS concentration illustrating improved AuNP stability.

15. SERS Measurement of 20 nm, 40nm, and 80 nm AuNP – Sc-MBs (3 µM)

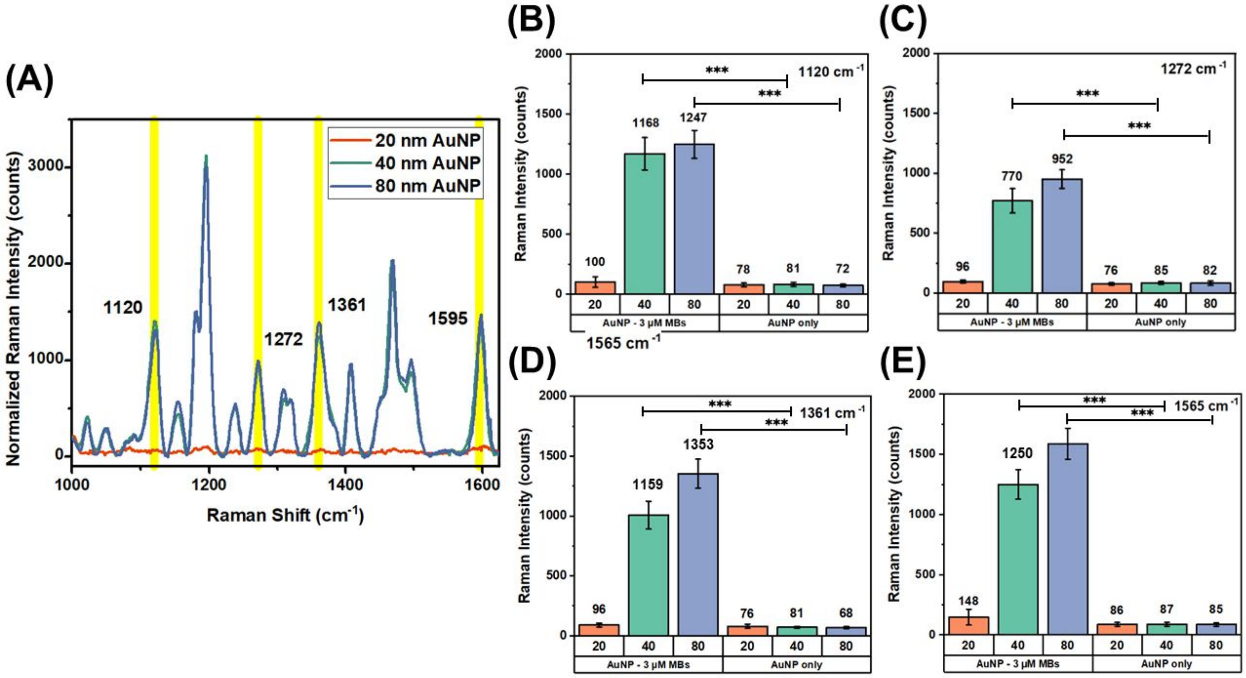

Figure S13. (A) SERS measurement comparison of Sc-MBs -AuNP with varying size ranging from 20, 40, and 80 nm AuNP respectively. A fixed 3 µM of initial DNA hairpin Cy5 (Sc-MBs) was loaded on the surface of the AuNP. Cy5 characteristic peaks were present in all AuNP of varying sizes, including ~1120 cm^{-1}, ~1272 cm^{-1}, ~1361 cm^{-1}, and ~1595 cm^{-1}. (B-E) illustrates the Raman intensity counts at each characteristic peak of AuNPs with varying diameters. Each experiment was repeated 3 times.

Table S11. The table illustrates the of SDS, NaCl, and oligonucleotide volume to 100 µL of AuNPs solution with the corresponding optical density purchased from the manufacturer before the freeze-assisted immobilization process.

Sample	***Diameter***	***Optical Density***	***17.6 mg/mL SDS (µL)***	***Initial 1M NaCl (µL)***	***Oligonucleotide***	***Oligonucleotide Concentration (µM)***	***Volume of Oligonucleotide (µL)***	***1 M NaCl during thawing (µL)***
1	*20 nm*	*10*	*13.2*	*3.5*	*MBs*	*3*	*6*	*10*
2	*40 nm*	*10*	*4.4*	*3.5*	*MBs*	*3*	*6*	*10*
3	*80 nm*	*10*	*4.4*	*3.5*	*MBs*	*3*	*6*	*10*

16. SERS Measurement as a function of initial Sc-MBs added in the reaction illustrating effects of packing densities (OD ~2.5)

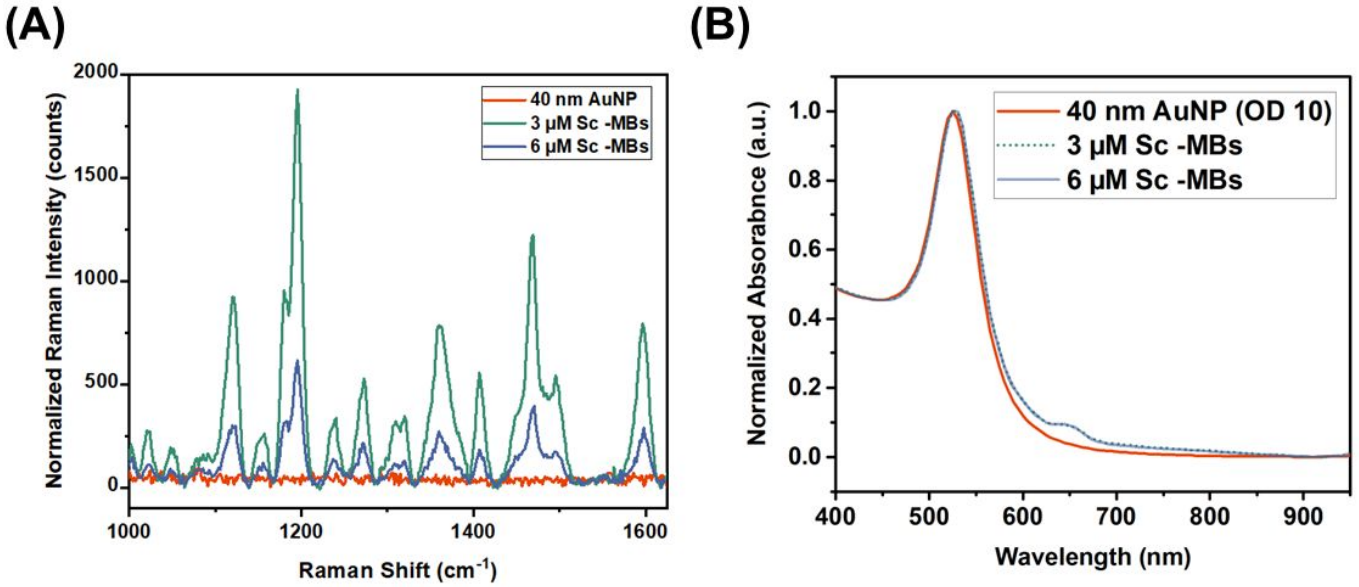

Figure S14. (A) SERS measurement comparison of Sc-MBs -AuNP with varying initial Sc-MB addition depicting the effects of packing densities on the AuNP surface. As reported in the nanodrop measurements, a total of oligonucleotide loading of 763 ng was measured for an initial addition of 3 μM of Sc-MBs while a total of 1165 ng was measured for 6 μM of Sc-MBs. (B) UV-Vis measurement of 3 μM of Sc-MBs and 6 μM of Sc-MBs conjugated AuNPs**.** Each experiment was repeated three times.

17. ζ-Potential Measurements of 20 nm, 40nm, and 80 nm AuNP – Sc-MBs (3 μM)

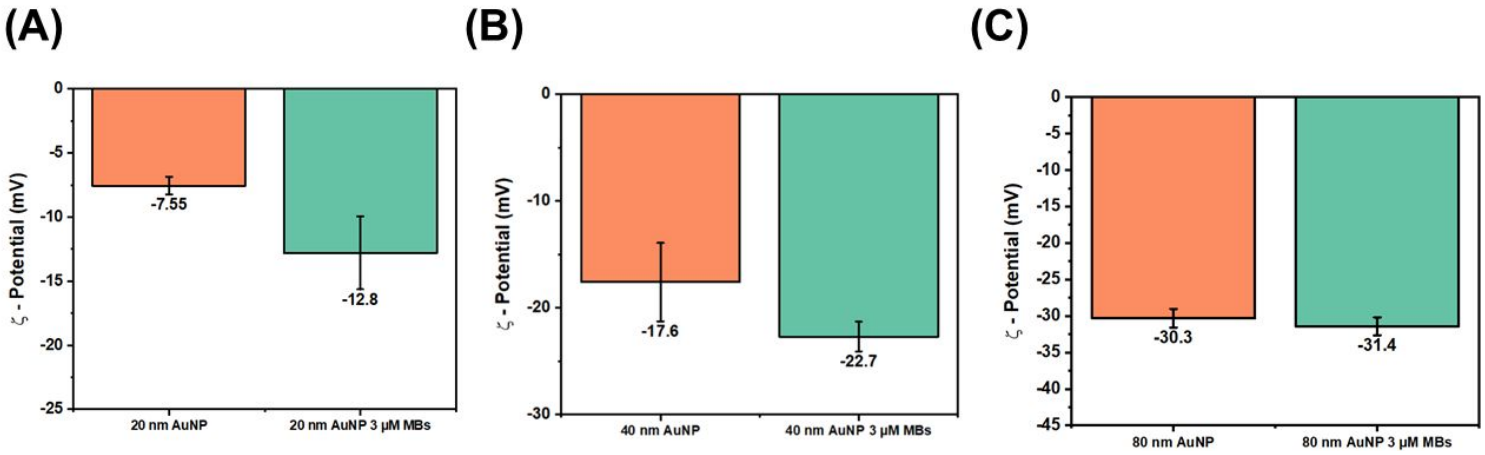

Figure S15. (A-C) ζ- potential measurements of 20, 40, and 80 nm AuNP before and after Sc-MBs immobilization on the AuNP surface. Both AuNP and Sc-MB-modified AuNPs were negatively charged, and immobilization of higher DNA densities should result in a more negatively charged surface. Our data suggests that the DNA loading made it slightly negative for 40 and 80 nm AuNPs. While 20-nm AuNPs exhibited a slightly more negative surface charge change. Each experiment was repeated three times.

18. Sc - MBs Loading Quantification using a Nanodrop Spectrophotometer

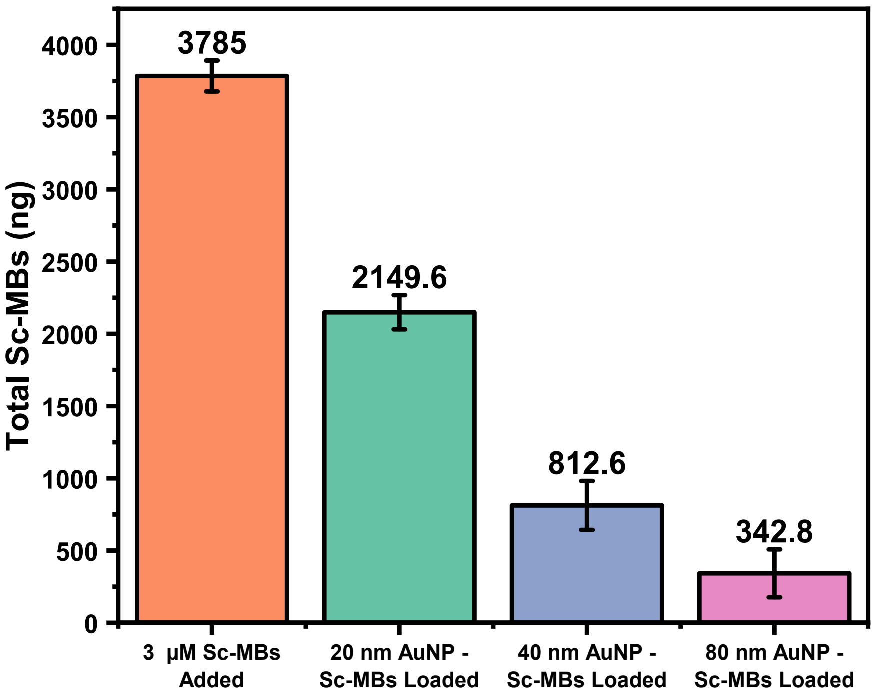

Figure S16. Nanodrop measurements of Sc-MBs added to the 100 µL AuNP solution with SDS and NaCl before freezing and the amount loaded after Sc-HPs immobilization (washed twice). Comparison of Sc-MBs loaded with respective sizes of 20, 40, and 80 nm. The nanodrop readings were all compared for the supernatant obtained after the DNA Hairpin-Cy5 (Sc-MBs) immobilization on the surface. The measurements confirm the successful loading of the Sc-MBs on the surface of the AuNPs. Each experiment was repeated three times.

19. Characterization of Sc-MBs co-immobilization with SH-mPEG using the modified freeze-assisted conditions.

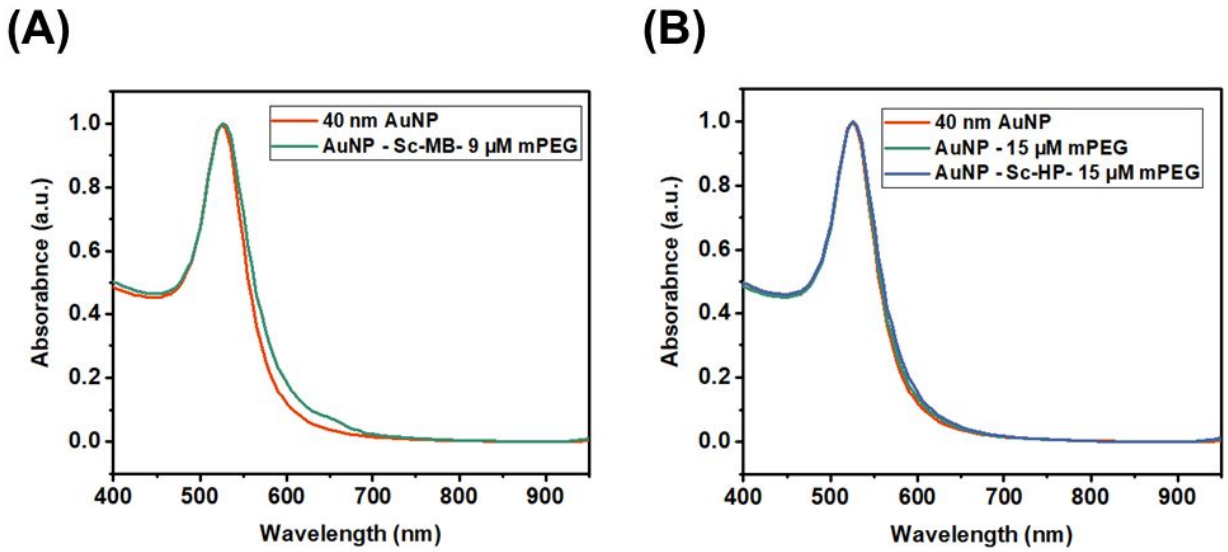

Figure S17. Normalized UV-Vis spectra illustrating successful SH-mPEG (MW:2000 Da) and Sc-MB/HP co-immobilization during the modified freeze-assisted functionalization. (A) illustrates the spectra after co-immobilization with 3 µM of Sc-MBs with 10 µL of 9 µM of SH-MPEG. The small peak in the ~650 nm corresponds to the absorbance of the Cy5 dye tethered on the Sc-MB sequence. **(**B). illustrates the spectra after co-immobilization of 6 µM of Sc-HPs and 10 µL of 15 µM of SH-mPEG during the modified freeze assisted functionalization. Each experiment was repeated three times.

20. Characterization of Sc-HP-mPEG post freeze-assisted immobilization via NaCl tolerance test.

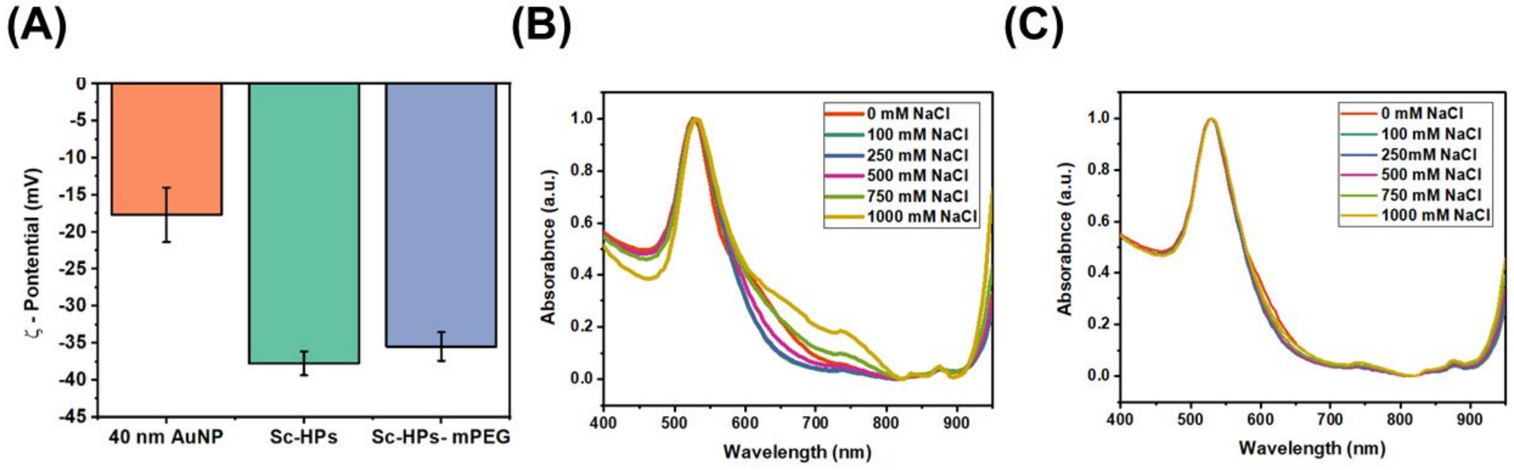

Figure S18. (A) ζ – potential measurements indicating successful Sc-HP immobilization and a slight positive shift after SH-mPEG post-immobilization. To verify that the mPEG was successfully conjugated on the surface, its protective properties were evaluated using a NaCl tolerance test by spiking NaCl solution incrementally and evaluating its stability**.** (B) Illustrates the normalized UV-Vis spectra of 3 μM Sc-HPs AuNP conjugates and its tolerance under varying salt conditions from 0 to 1000 mM NaCl. (C) Illustrates the stabilization of the 3 μM Sc-HPs after modification with SH-mPEG, confirming successful conjugation due to enhanced protective properties. Each experiment was repeated three times.

21. Cy5 tagged DNA Target Standard Curve and Supernatant Samples

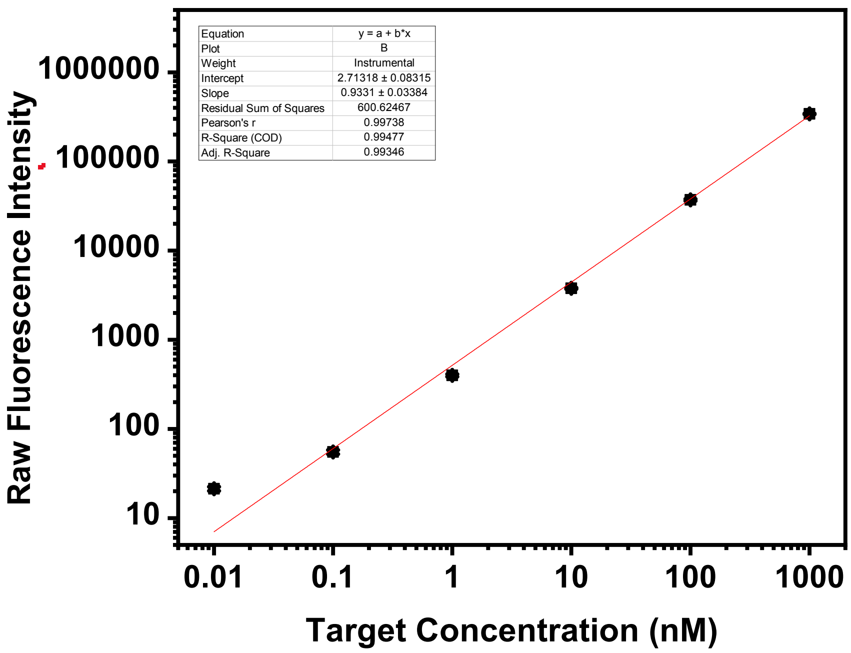

Figure S19. DNA target conjugated with the Cy5 standard curve and associated fluorescence intensity. The fluorescence intensity of the Cy5-tagged DNA target sequence was measured with a 10-fold dilution factor from 0.01 nM to 1000 nM to obtain a linear trend. These were the stock solutions used for the hybridization experiment.

22. Cy5 tagged DNA Target Standard Curve vs Supernatant of Immobilized DNA Target to 40 nm AuNP – Hairpin Oligos (3 µM)

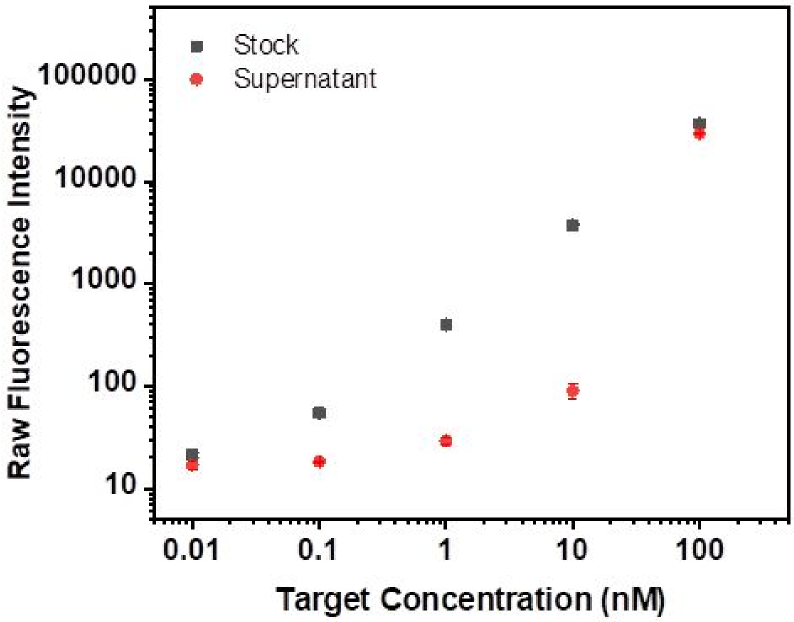

Figure S20. The plot shows the raw fluorescence intensity in relation to the target concentration (nM). Each experiment was repeated three times.

23. Raw Fluorescence Intensity of washed 40 nm AuNP – Hairpin Oligos (3 μM)

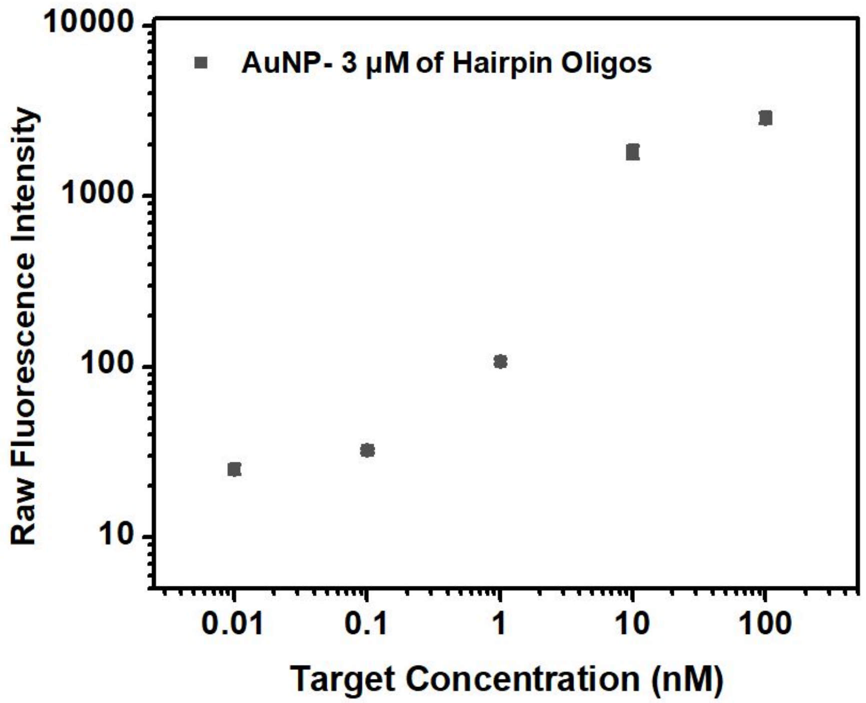

Figure S21. The plot shows the calculated raw fluorescence intensity corresponding to the fluorescently tagged DNA target sequence loaded onto the Sc-HP functionalized 40 nm AuNPs.

References:

1. Liu, B. & Liu, J. Freezing Directed Construction of Bio/Nano Interfaces: Reagentless Conjugation, Denser Spherical Nucleic Acids, and Better Nanoflares. *J. Am. Chem. Soc.* **139**, 9471–9474 (2017).
